# PSMC5 Orchestrates an Immunosuppressive Niche and Metastasis in Colorectal Cancer via SMURF1-Mediated K11-Linked Ubiquitination of METTL14

**DOI:** 10.7150/ijbs.130350

**Published:** 2026-05-11

**Authors:** Yi Zhang, Xiao Yang, Hua Zhong, Chengsheng Ding, Ximo Xu, Yanfei Shao, Bin Sun, Wendong Zhang, Junjun Ma, Bo Feng, Minhua Zheng, Zirui He

**Affiliations:** 1 Department of General Surgery, Ruijin Hospital, Shanghai Jiao Tong University School of Medicine, Shanghai 200025, China.; 2 Shanghai Institute of Digestive Surgery, Ruijin Hospital, Shanghai Jiao Tong University School of Medicine, Shanghai 200025, China.; 3 Department of General Surgery, Loujiang New City Hospital of Taicang (Taicang Branch of Ruijin Hospital Affiliated with Shanghai Jiao Tong University School of Medicine), Suzhou 215400, China.; 4 Department of General Surgery, Shanghai General Hospital Shanghai Jiao Tong University School of Medicine, Shanghai 200080, China.

**Keywords:** Colorectal cancer, PSMC5, METTL14, Metastasis, Ubiquitination, Tumor immune microenvironment

## Abstract

Colorectal cancer (CRC) metastasis requires coordination between tumor-intrinsic programs and the surrounding microenvironment, yet how proteasomal regulation intersects with the epitranscriptome in this process remains unclear. Here, analyses of TCGA, GEO, and an institutional cohort of 146 CRC patients identified PSMC5 upregulation as associated with metastatic progression and poor prognosis. Mechanistically, PSMC5 promoted SMURF1-dependent K11-linked ubiquitination of METTL14 at K263, leading to METTL14 destabilization, global m⁶A remodeling, and activation of EMT-associated malignant phenotypes. Rescue experiments further supported METTL14 as a functional downstream effector of PSMC5. Integrative single-cell, spatial transcriptomic, and multiplex immunofluorescence analyses showed that PSMC5-high epithelial states were associated with spatially organized “regulatory islands,” defined here as PSMC5-high epithelial nests with peripheral Treg and M2 enrichment together with relative CD8⁺ T-cell exclusion. *In vivo*, SMURF1 silencing restored METTL14 expression and attenuated PSMC5-driven tumor growth and lung metastasis. Collectively, these findings define a PSMC5/SMURF1/METTL14 axis that links proteasomal regulation to epitranscriptomic remodeling and metastatic progression in CRC, and identify this pathway as a candidate therapeutically actionable axis.

## Introduction

Colorectal cancer (CRC) remains a major global health burden, accounting for approximately 1.9 million new cases and 935,000 deaths annually worldwide 1. The burden of early-onset CRC is rising, with increasing incidence and mortality in individuals aged ≤ 50 years 2, 3. About 20% of patients present with synchronous metastases, and up to 50% of those initially diagnosed with localized disease eventually develop metastatic CRC (mCRC). Although survival in mCRC has improved over the past two decades, outcomes remain substantially worse than those in non-metastatic disease 4. Despite advances in molecularly guided therapies, the mechanisms driving CRC invasion and dissemination remain unclear 5.

CRC metastasis is driven by both tumor-intrinsic programs and extrinsic cues from the tumor microenvironment (TME) 6, 7. Among these processes, epithelial-mesenchymal transition (EMT) is a central event that promotes motility, invasion, and metastatic competence 8-10. However, the protein-level and post-translational regulation of metastasis drivers remains poorly defined.

Ubiquitination is a central post-translational modification that controls protein stability 11. The ubiquitin-proteasome system (UPS) uses an E1/E2/E3 cascade to assemble polyubiquitin chains and the 26S proteasome to degrade substrates 12. It also regulates transcription factors, epigenetic regulators, and major oncoproteins 13. UPS function is essential for normal colonic epithelium, and its dysregulation contributes to CRC development 14. The 26S proteasome is formed by the 20S catalytic particle and either one or two of the 19S regulatory units 15, 16. PSMC5 is an AAA⁺ ATPase subunit of the 19S particle that helps recognize and translocate ubiquitinated substrates 17. PSMC5 is elevated in multiple tumor types, including malignancies of the pancreas and head-and-neck 18, 19, and correlates with worse survival in breast cancer 20. In CRC, PSMC5 overexpression promotes proliferation and metastasis and correlates with components of the m⁶A machinery 21. However, how PSMC5 selectively drives metastatic progression remains unclear.

RNA methyltransferases have emerged as UPS substrates of interest. N⁶-methyladenosine (m⁶A) is the most abundant internal RNA modification in eukaryotes 22. METTL14 is a core “writer” for m⁶A deposition on nuclear RNA 23. Global m⁶A decreases when METTL14 is deleted in mouse embryonic stem cells 24. Across cancers, METTL14 often suppresses progression. The loss of METTL14 promotes lung metastasis in renal cancer 25. METTL14 mutations enhance endometrial tumor growth 26 and low METTL14 serves as a strong predictor of unfavorable prognosis, evidenced by shorter recurrence-free survival and by its correlation with metastatic progression in hepatocellular carcinoma 27. In CRC, most studies focus on downstream targets. METTL14 knockdown reduces m⁶A on SOX4, XIST and others, increasing their expression and driving metastasis 28, 29. By contrast, how METTL14 protein stability is regulated in CRC is unknown. Components of the m⁶A machinery can be controlled by post-translational modifications, including ubiquitination, SUMOylation, and acetylation 30. Clarifying METTL14's post-translational control may therefore reveal new mechanisms of CRC metastasis.

SMAD ubiquitination regulatory factor 1 (SMURF1) is a HECT-type E3 ligase 31. It is broadly expressed and ubiquitinates many substrates that regulate development and cancer cell invasion 32, 33. SMURF1 overexpression associates with poor prognosis in several cancers, including glioblastoma and colon cancer 34, 35. These observations nominate SMURF1 as a plausible candidate E3 ligase involved in the regulation of METTL14 stability in CRC.

Beyond cell-intrinsic metastatic programs, the immunosuppressive tumor microenvironment is increasingly recognized as an important determinant of CRC progression and dissemination 6, 36. What intrinsic drivers such as PSMC5 shape this spatial immune landscape remains unclear. Emerging single-cell and spatial transcriptomic technologies now provide an opportunity to resolve how PSMC5 shapes the spatial immune architecture of CRC.

Here, we show that PSMC5 promotes SMURF1-dependent ubiquitin-mediated degradation of METTL14 in CRC cells. Loss of METTL14 enhances EMT and metastatic traits. By integrating biochemical, functional, single-cell, and spatial transcriptomic analyses, we identify a PSMC5/SMURF1/METTL14 axis that promotes metastatic progression and is associated with a distinct immune contexture in CRC, highlighting PSMC5 as a potential biomarker and candidate therapeutic target.

## Methods

### Pan-cancer differential expression analysis

The expression pattern of PSMC5 across multiple cancer types was evaluated through the TIMER3.0 web interface. Variations in transcriptomic profiles across tumor samples and adjacent unaffected tissues were evaluated via Wilcoxon rank-sum analysis. Findings were deemed statistically meaningful whenever the associated P value fell short of 0.05.

### Correlation analysis of gene expression

Correlations between PSMC5 and selected genes were analyzed using transcriptomic datasets from the aforementioned sources. Pearson correlation coefficients were derived to assess correlation magnitude, while corresponding p-values were employed to establish the level of statistical relevance.

### Survival analysis

The prognostic significance of gene expression was investigated utilizing clinical plus RNA-seq data from the TCGA cohort. Following stratification by expression levels, we generated Kaplan-Meier plots to visualize overall survival trends. Discrepancies in clinical outcomes among the defined subgroups were rigorously tested using log-rank statistics.

### Functional enrichment analysis

Functional annotation of differentially expressed transcripts was executed via KEGG pathway analysis within the clusterProfiler environment. We utilized an FDR threshold of less than 0.05 to determine which metabolic or signaling pathways were statistically overrepresented.

### MeRIP library construction and sequencing

Total RNA was extracted and fragmented (~200 nt) using reagents from CloudSeq, Shanghai, China. RNA fragments were immunoprecipitated with anti-m⁶A antibody (Millipore, USA) following the manufacturer's guidelines. Libraries were then generated from both the input and the immunoprecipitated RNA fractions using Illumina's TruSeq kit for RNA sample prep, which involved capture by protein A/G magnetic beads, requisite washes, and subsequent elution. Agilent's 2100 Bioanalyzer system was then employed to validate the library quality. Finally, paired-end sequencing was conducted utilizing Illumina's NovaSeq 6000 platform.

### MeRIP-seq data processing

Initial sequence reads were processed for adapter removal and low-quality base trimming using Cutadapt. The resulting clean reads were subsequently aligned to the reference genome (hg38) with HISAT2 v2.0.4. MACS2 was employed for peak identification, while diffReps detected differentially methylated regions. Peaks within exonic segments of mRNAs, lncRNAs, and circRNAs were annotated, and enriched genes were subjected to KEGG pathway analysis.

### RNA sequencing

Total RNA was extracted from the indicated cells using Trizol reagent (Invitrogen, Carlsbad, CA, USA). mRNA was purified, fragmented, and used for strand-specific library construction with the GenSeq® mRNA Purification Kit and GenSeq® Directional RNA Library Prep Kit (GenSeq, Inc., Shanghai, China). After executing 150-bp paired-end sequencing, the resulting data were cleaned using fastp and aligned to the hg38 genome via HISAT2. FeatureCounts served to estimate gene expression levels, after which edgeR was employed to discern significant mRNA variations (|log2FC| ≥ 1, P < 0.05). Finally, the biological significance of these genes was explored through Gene Ontology and KEGG pathway annotation.

### Human samples and tissue microarray

Paired colorectal tumor specimens and non-tumorous counterparts (taken > 5 cm from the tumor margin) were obtained from patients undergoing resection at Ruijin Hospital, Shanghai Jiao Tong University School of Medicine. No individuals had received treatment before surgery. Key clinicopathological data, including patient age, sex, TNM classification, and recurrence status, were documented. Freshly excised tissues underwent prompt snap-freezing within liquid nitrogen and were thereafter archived at -80 °C. Concurrently, specimens preserved in formalin were subjected to paraffin infiltration and embedding. A tissue microarray (TMA) was subsequently built, comprising 151 paired CRC and nearby tissues, for analysis by immunohistochemistry (IHC). After exclusion of five cases without evaluable follow-up information, 146 patients were included in subsequent clinicopathological correlation and Kaplan-Meier survival analyses. Cox regression analyses were performed using complete-case data, resulting in 144 patients included in the univariate and multivariate Cox analyses. Written informed consent was obtained from all participants, and the study was approved by the Ruijin Hospital Ethics Committee (No.2020384).

### Immunohistochemistry and clinicopathological analysis

The TMA slides were initially treated with deparaffinization and rehydration. Subsequently, a thermal-induced antigen retrieval protocol (Tris-EDTA, pH 9.0) was followed. A 3% H₂O₂ solution was applied to neutralize intrinsic peroxidase activity, followed by a subsequent blocking using 5% BSA solution. Immunohistochemical staining involved treating samples with antibodies for PSMC5 (1:500), SMURF1 (1:200), and METTL14 (1:1000) under cold conditions (4 °C) for 12-16 h. Signal amplification was subsequently performed using Dako EnVision™ reagents, with DAB serving to visualize the immunoreactivity. The experimental procedure concluded with hematoxylin counterstaining, dehydration, and final mounting. Quantitative analysis was executed using ImageJ, while the DAB signal was isolated via color deconvolution. The resultant staining intensity was stratified into three tiers: weak, moderate, or strong, and an H-score was calculated as Σ(intensity × % positive). Finally, the resulting scores were applied for correlation analyses with clinicopathological parameters and for survival analyses, with detailed statistical methods described in the Statistical analysis section.

### Cell culture and transfection

Human-derived HEK293T, RKO, and HCT116 cells (ATCC, Manassas, VA) were propagated in a controlled incubator (5% CO₂, 37 °C). The basal media, consisting of DMEM or RPMI-1640, were modified with 10% heat-inactivated FBS and standard antibiotics. All cell culture procedures adhered to established protocols for maintaining humidified growth environments. HCT116 and RKO are commonly used CRC cell lines with MSI-high molecular features and CMS1-like characteristics 37. Lentiviral vectors for PSMC5 overexpression, METTL14 overexpression, SMURF1 knockdown, METTL14 knockdown, and CKIP-1 knockdown were purchased from Shanghai Genechem Co., Ltd. Stable cell pools were generated by antibiotic selection. For dual-gene manipulation, orthogonal antibiotic markers were used. Corresponding empty lentiviral vectors served as negative controls. For the ectopic expression experiments, we acquired plasmids from Shanghai Genechem Co., Ltd., which encompassed Flag-METTL14 variants (WT and three specific lysine-to-arginine substitutions), Myc-labeled PSMC5, and SMURF1. The ubiquitination machinery was studied via HA-ubiquitin plasmids harboring different lysine linkages (K6 through K63). Following the manufacturer's guidance, these constructs-or a blank Flag vector for control purposes-were introduced into cells using a specialized lipid-based delivery system.

### Cell proliferation and colony formation assays

To quantitatively measure cellular proliferation, 3 × 10³ cells/well were inoculated into 96-well microplates. Each experimental condition comprised sextuplicate technical replicates, and the entire protocol was executed in triplicate on separate occasions. To evaluate growth kinetics, CCK-8 reagent (Meilunbio) was introduced to the wells at 24-hour intervals up to 96 hours. We quantified cellular viability by measuring 450-nm spectrophotometric absorbance. For the long-term survival assay, we inoculated 400 cells into each 6-well plate and maintained them in 10% FBS growth medium for a fortnight. The colonies were eventually immobilized, stained with a 0.1% crystal violet concentration for half an hour, and the number of macroscopically visible foci was recorded.

### Wound healing and transwell assays

In the wound-healing protocol, a confluent layer of cells was wounded using a 10 μL pipette tip, rinsed with PBS, and incubated in serum-free medium. Images were captured at set intervals, and migration was expressed as (A₀ - Aₙ) / A₀ × 100(%). Invasion and migration capacities were assessed by seeding cells within upper chambers-either bare or Matrigel-coated-using serum-depleted media. A 20% fetal bovine serum gradient was established in the underlying wells to induce movement. After 48 hours, the migrated population was fixed in a 4% methanol solution and visualized with 0.5% crystal violet. We then determined cell numbers through microscopic observation across three independent replicates to ensure statistical consistency.

### qRT-PCR

To evaluate mRNA levels, Trizol-based extraction was performed to obtain total RNA, which was then used as a template for cDNA synthesis (1 μg per reaction) via Vazyme's HiScript III RT SuperMix. Real-time quantitative analysis was conducted using the ChamQ Universal SYBR qPCR kit. Transcriptional variations were quantified through the 2^-ΔΔCt^ formula, with GAPDH serving as the internal reference for normalization. All oligonucleotide sequences utilized for these assays are documented in the supplementary materials.

### Western blot assay

Cellular disruption was executed for 30 min under cold conditions (on ice) employing a lysis buffer augmented with a cocktail of protease and phosphatase inhibitors. The resulting lysates were resolved by SDS-PAGE gel. The electroblotted PVDF membranes were then subjected to a blocking protocol using 5% BSA. Probing with the designated primary antibodies was conducted for 16 hours at 4 °C. After proper washing, horseradish peroxidase-conjugated secondary antibodies were used to incubate the designated membranes at room temperature. A detailed inventory of the antibodies employed for this immunoblotting is provided in the Supplementary Information.

### LC-MS/MS

The interactome of PSMC5 was interrogated by subjecting cellular lysates to immunoprecipitation, utilizing either an anti-PSMC5 antibody or a non-targeting IgG acting as an isotype control. The immunoprecipitated material was subjected to reduction, alkylation, and tryptic digestion. Subsequent proteomic analysis by LC-MS/MS was executed using a timsTOF Pro (Bruker) mass spectrometer interfaced with a nanoElute LC system. Data acquisition was in DIA mode, and identification used DIA-NN against the Homo sapiens UniProt database (1% FDR). For METTL14 ubiquitination profiling, digested peptides were enriched using anti-K-ε-GG resin (CST) and analyzed under the same LC-MS/MS conditions. Spectra were processed via Spectronaut (Biognosys), retaining peptides with localization probability > 0.75.

### Immunofluorescence staining

Cells on coverslips were fixed, permeabilized (Servicebio G1204), blocked (3% BSA or serum), and incubated overnight with antibodies against METTL14, PSMC5, or SMURF1. Following the application of HRP-linked secondary antisera and TSA-mediated amplification, DAPI was used for nuclear counterstaining. We secured the samples in an antifade reagent to preserve fluorescence. Finally, microscopic observation and data acquisition were performed on a Nikon Eclipse C1 confocal scanning system.

### Co-immunoprecipitation

Co-IP experiments were conducted using the rProtein A/G Magnetic IP Kit (ACE Biotechnology). Protein lysates (500-1000 µg) were incubated at 4 °C with 2-10 µg of the designated antibodies, which were subsequently captured by magnetic beads. Following washes, bound material was released in loading buffer and examined via SDS-PAGE and Western blotting.

### METTL14 ubiquitylation assay

Cells were incubated with MG132 (15 μM) for 6 hours beforehand, then proper lysation and incubation with an anti-METTL14 antibody were performed. Immunoprecipitates were captured on protein A/G beads, washed, and analyzed by immunoblotting to assess METTL14 ubiquitination levels.

### MG132 treatment and cycloheximide chase assay

For proteasome inhibition, the indicated cells were treated with MG132 (5 μM) and harvested at the specified time points for immunoblotting. For protein stability analysis, cells were treated with cycloheximide (CHX, 50 μg/mL) to inhibit *de novo* protein synthesis and collected at the indicated time points. Whole-cell lysates were then prepared for immunoblotting, and GAPDH was used as the loading control.

### *In vitro* ubiquitination assay

Purified recombinant human SMURF1 (S254-381AG) and full-length human METTL14 (M334-30G) were incubated in a 20 μL ubiquitination reaction containing E1, the indicated E2, ubiquitin, Mg²⁺-ATP, and reaction buffer from the E2Select Ubiquitin Conjugation Kit at 37°C for one hour. Following the addition of 5× SDS loading buffer and subsequent denaturation at high temperature, the processed samples were resolved via SDS-PAGE and transferred for immunoblotting. We employed a universal anti-ubiquitin antibody to visualize overall ubiquitination, alongside a K11-specific linkage-selective antibody (clone MABS107-I) to pinpoint K11-type modifications.

### Xenograft and lung metastasis models

Four-week-old male BALB/c nude mice were obtained from the Shanghai Laboratory Animal Research Center (Shanghai, China) and maintained under specific pathogen-free conditions. HCT116 cells stably transduced with the indicated lentiviral vectors were injected into the right inguinal fat pad. For the primary xenograft experiment, mice were assigned to four groups receiving NC, PSMC5-OE, SMURF1-sh, or PSMC5-OE+SMURF1-sh cells (n = 9 per group). For the rescue experiment, mice were assigned to three groups receiving NC, SMURF1-sh, or SMURF1-sh+METTL14-sh cells (n = 6 per group). In both experiments, 5×10⁶ cells were implanted per mouse, tumor length and width were measured at regular intervals, and mice were euthanized when tumor volume approached the humane endpoint of 2000 mm³. Xenograft tumors were then excised and weighed. For the CKIP-1 experiment, mice were assigned to two groups receiving PSMC5-OE+CKIP-1-NC or PSMC5-OE+CKIP-1-sh cells (n = 4 per group). Each mouse was injected with 2×10⁶ cells and euthanized 2 weeks after inoculation, after which tumors were excised and weighed. Excised tumors were processed for hematoxylin-eosin staining and, where indicated, immunohistochemical analysis.

To establish the pulmonary metastasis model, HCT116 cells already harboring stable expression of NC, PSMC5-OE, SMURF1-sh, or the PSMC5-OE+SMURF1-sh combination were thereafter transduced with a luciferase-expressing lentivirus (Shanghai GeneChem Co., Ltd., Shanghai, China). Four-week-old male BALB/c nude mice were apportioned into four cohorts (n = 9 per group), and each animal was administered 1 × 10⁶ cells through the tail vein. For the lung metastasis model, the experimental endpoint was set at 28 days post-injection, based on established protocols and previous studies. At 28 days following inoculation, the mice were anesthetized with isoflurane, and 100 μL of D-luciferin was given via intraperitoneal injection. To track cancer cell dissemination, bioluminescent signals were acquired using the *In vivo* Imaging System (IVIS) Spectrum (PerkinElmer). Investigators were blinded to group allocation during model establishment, sample collection, and statistical analysis. All protocols involving animal husbandry and experiments received formal approval from the Institutional Animal Care and Use Committee of Ruijin Hospital, Shanghai Jiao Tong University School of Medicine.

### Single-cell analysis

Four colorectal cancer tissue samples were obtained from Ruijin Hospital. After removal of adipose tissue and blood vessels, tissues were dissociated into single-cell suspensions by enzymatic digestion and mechanical disruption. Single-cell libraries were prepared using the 10x Chromium platform and sequenced on a NovaSeq 6000 system. Raw data were converted to FASTQ files, subjected to quality control, and aligned to the human reference genome. Subsequent analyses were performed in R (version 4.4.1) with Seurat (version 4.4.0) for filtering, normalization, clustering, and cell-type annotation, and Harmony (version 1.2.1) was used for batch correction. Cluster-specific marker genes were identified, and metabolic pathway activity was further analyzed.

### Spatial transcriptomics

Spatial transcriptomic data were downloaded from GEO, including five colorectal cancer tumors (GSM7058756-GSM7058759 and GSM7573205) and one normal colonic tissue sample (GSM8286350), derived from GSE225857 and GSE236698. Spatial data were processed using Scanpy, with mitochondrial genes filtered prior to downstream analysis. After quality control and normalization, batch effects across slides were corrected using Harmony (version 1.2.1). Dimensionality reduction and spot clustering were then performed. To infer the spatial distribution of cell types, Cell2location was applied using our single-cell RNA-seq dataset as the reference. Spatial cell-cell interaction patterns within a 200-μm range were further analyzed using MISTY. Bioinformatic analyses were performed in R (version 4.4.1) where applicable.

### Multiplex immunofluorescence

Formalin-fixed paraffin-embedded primary colorectal tumor sections from the institutional cohort were subjected to TSA-based multiplex immunofluorescence staining. The specimens included primary tumor sections from two groups of patients (4 cases per group): stage I CRC patients without metastatic lesions and stage IV CRC patients with metastatic disease. After deparaffinization, rehydration, endogenous peroxidase blocking, antigen retrieval in Tris-EDTA buffer (pH 9.0), and 5% BSA blocking, sections were sequentially incubated with primary antibodies, HRP-conjugated secondary antibody, and fluorophore-conjugated TSA reagents. Antigen retrieval was repeated between staining cycles. The antibodies used were PSMC5, CD206, FOXP3, CD4, and CD8, with fluorophores assigned as Alexa Fluor 488, Cy7, 594, Cy5, and Cy3, respectively. Nuclei were counterstained with DAPI, and images were acquired for subsequent analysis.

### Statistical analysis

All computational analyses were conducted within the R (v4.4.1) and Prism 10.1.0 environments. We analyzed the association between categorical clinical data and protein expression status via Fisher's exact method. The prognostic value of target genes was determined using Kaplan-Meier survival plots alongside Cox regression analysis. Typically, data are expressed as mean ± SD, reflecting at least three separate experimental runs. Discrepancies between two cohorts were validated using independent t-tests, while larger datasets were scrutinized via ANOVA combined with Tukey's multiple comparison procedure. For omics-related or nonparametric statistics, specific methodologies are provided in the corresponding legends. Throughout the study, two-sided tests were used, and p-values beneath 0.05, 0.01, 0.001, or 0.0001 were assigned different levels of statistical importance.

## Results

### PSMC5 remodels the m⁶A epitranscriptome via METTL14 suppression and predicts poor prognosis

**Figure 1:**
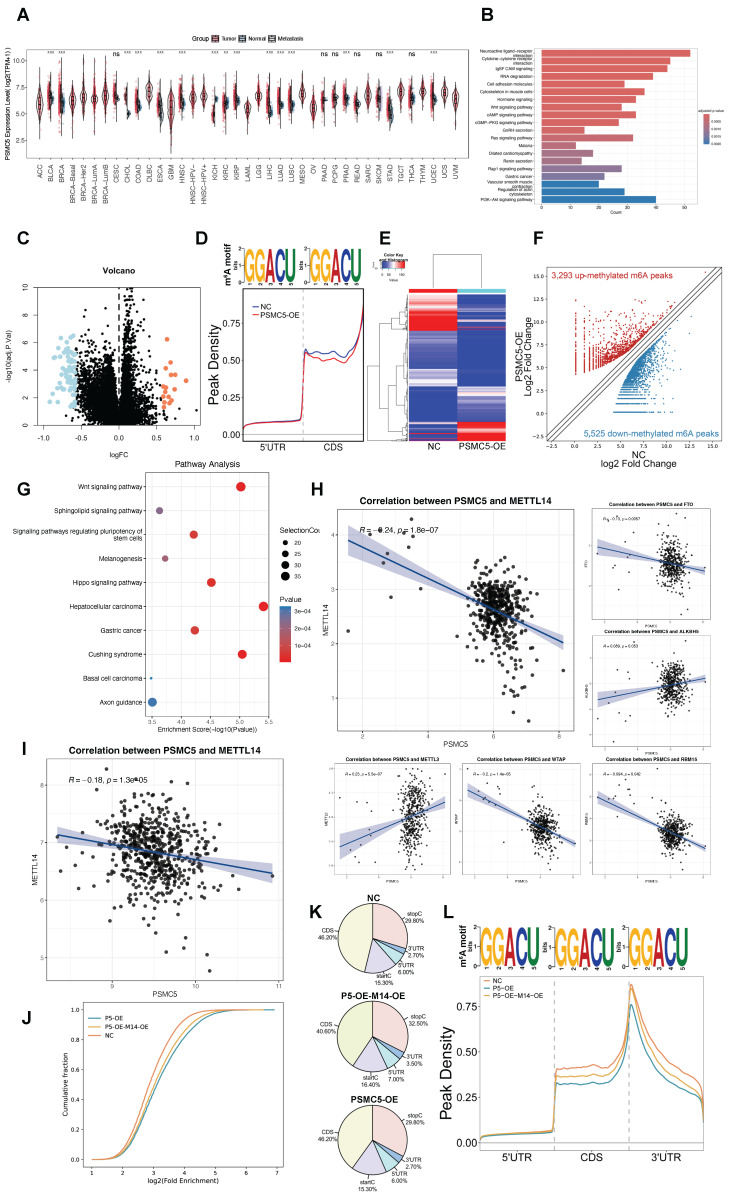
** PSMC5 is upregulated in CRC and associates with a remodeled m⁶A landscape and poor prognosis. (A)** Pan-cancer analysis of PSMC5 expression in tumor, matched normal, and metastatic tissues. **(B)** KEGG pathway enrichment of differentially expressed genes between PSMC5-high and PSMC5-low colorectal cancer samples. **(C)** Volcano plot showing differentially expressed genes between PSMC5-high and PSMC5-low groups. Upregulated genes are shown in orange and downregulated genes in blue. **(D)** Consensus m⁶A motif and metagene distribution along 5′UTR-CDS-3′UTR in PSMC5-overexpressing (PSMC5-OE) versus control (NC) cells. **(E)** Heatmap of MeRIP-seq peak intensities comparing NC and PSMC5-OE cells. **(F)** Volcano plot of differential m⁶A peaks in PSMC5-OE versus NC. **(G)** KEGG enrichment of genes harboring altered m⁶A peaks upon PSMC5 overexpression. **(H)** Correlation analyses between PSMC5 and m⁶A regulators (METTL14, FTO, ALKBH5, METTL3, WTAP, RBM15) in CRC samples from TCGA dataset. **(I)** Validation of PSMC5-METTL14 correlation in the independent GSE39582 cohort. **(J)** Cumulative distribution of log₂ enrichment for NC, PSMC5-OE (P5-OE), and PSMC5-OE+ METTL14-OE (P5-OE-M14-OE) cells. **(K)** m⁶A peak profiles across transcriptional regions (5′UTR, CDS, start/stop codon vicinity, 3′UTR) in NC, P5-OE, and P5-OE-M14-OE cells. **(L)** Metagene peak density profiles across 5′UTR-CDS-3′UTR in NC, P5-OE, and P5-OE-M14-OE cells. Statistical analysis: Wilcoxon rank-sum test for (A); hypergeometric test for (B, G); empirical Bayes moderated t-statistics (limma) for (C); Pearson correlation analysis for (H, I). *P<0.05, **P<0.01, ***P<0.001, ****P<0.0001, ns, not significant.

To explore the role of PSMC5 in human cancer, we initially analyzed PSMC5 expression levels within the TCGA pan-cancer cohort. The data showed that PSMC5 was elevated across various cancer types, including colorectal (COAD and READ), liver (LIHC), and lung (LUAD) cancers, as well as several other malignancies, indicating a potential oncogenic role of PSMC5 (Fig. 1A). In colorectal cancer specifically, differential expression analysis between PSMC5-high and PSMC5-low samples identified a robust set of dysregulated genes, as illustrated by the volcano plot (Fig. 1C). KEGG enrichment of these PSMC5-related DEGs showed a prominent RNA degradation pathway together with multiple cancer-associated signaling cascades, including Wnt and PI3K-Akt and other carcinoma-related terms (Fig. 1B). This raised the possibility that PSMC5 might influence RNA metabolism. In line with our prior study showing that RNA metabolism mediated by the m⁶A machinery is a key mechanism of CRC metastasis and was predicted to intersect with PSMC5, we next asked whether PSMC5 reshapes the m⁶A landscape. As expected, MeRIP-seq assay revealed a global m⁶A remodeling in PSMC5-overexpressing cells, with 3,293 up-methylated and 5,525 down-methylated peaks identified (Fig. 1D-F).

**Figure 2:**
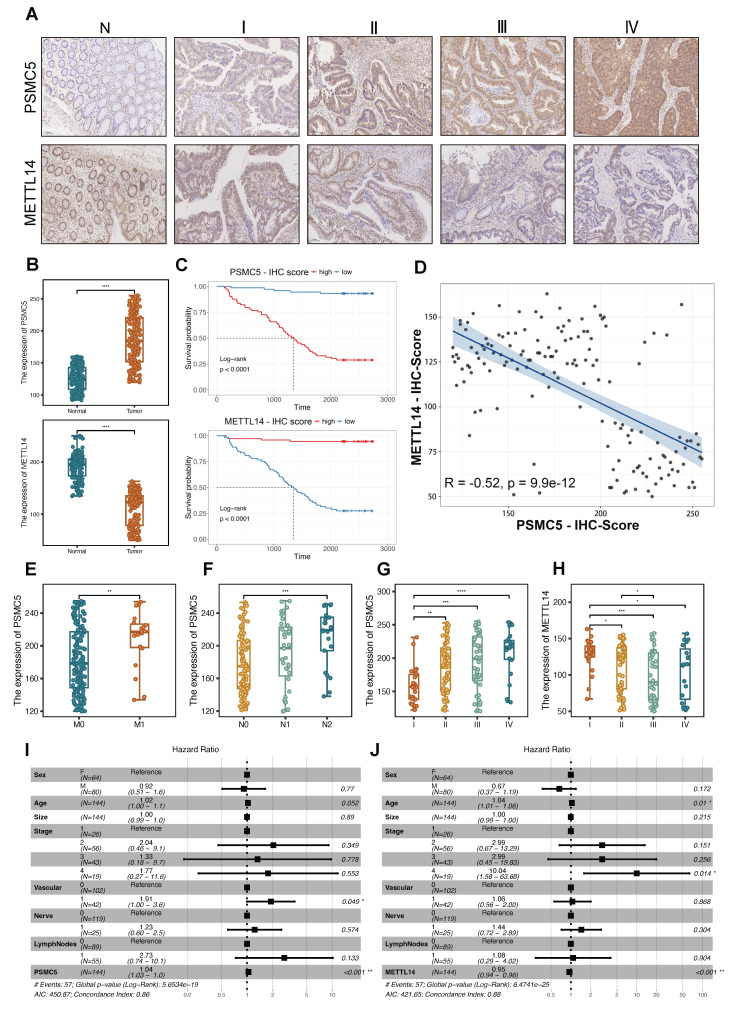
** Clinicopathological and prognostic outcome based on PSMC5 and METTL14 expression in CRC. (A)** Representative IHC images of PSMC5 and METTL14 in normal colorectal mucosa (N) and CRC stages I-IV. **(B)** Boxplots of IHC scores comparing tumor versus adjacent normal for PSMC5 (upper) and METTL14 (lower). **(C)** Kaplan-Meier overall-survival curves stratified by high versus low IHC scores for PSMC5 (top) and METTL14 (bottom) in our CRC cohort (n=146; log-rank p<0.0001 for both). **(D)** Scatterplot of paired PSMC5- and METTL14-IHC scores with linear fit; Pearson correlation is indicated (r=-0.52, p=9.9×10⁻¹²). **(E-G)** PSMC5 IHC scores stratified by M stage (M0/M1), N stage (N0/N1/N2), and overall AJCC stage (I-IV), respectively. **(H)** METTL14 IHC scores stratified by overall AJCC stage (I-IV). **(I-J)** Multivariable Cox proportional-hazards models showing hazard ratios (HR) and 95% CIs for PSMC5 (I) and METTL14 (J) with the indicated covariates. Statistical analysis: Wilcoxon rank-sum test for (B, E); log-rank test for (C); Pearson correlation analysis for (D); Kruskal-Wallis test with multiple-comparison correction for (F-H); Wald test from Cox proportional hazards regression for (I, J). *P<0.05, **P<0.01, ***P<0.001, ****P<0.0001, ns, not significant.

The differentially methylated transcripts were enriched in Wnt signaling and signaling pathways regulating pluripotency of stem cells, along with other cancer-related programs (Fig. 1G), and GO analysis further emphasized Wnt-related processes, ubiquitin transferase activities and synapse-associated cellular components (Fig. S1B-D). In parallel, RNA-seq analysis of PSMC5-overexpressing cells identified differentially expressed mRNAs enriched in pathways associated with extracellular matrix interaction, focal adhesion, integrin signaling, and cytoskeletal remodeling, supporting a more invasive cellular program (Fig. S1E). Importantly, PSMC5 expression inversely correlated with METTL14 (Pearson R = -0.24, p = 1.8 × 10⁻⁷; Fig. 1H), and this association was validated in an independent GEO cohort (GSE39582) (R = -0.18, p = 1.3 × 10⁻⁵; Fig. 1I). Rescue MeRIP-seq further showed that METTL14 co-expression partially restored the global m⁶A enrichment and peak distribution perturbed by PSMC5 (Fig. 1J-L). Consistently, when TCGA-CRC patients were stratified into four clusters according to combined PSMC5 and METTL14 expression, overall survival differed significantly among the groups (log-rank p = 0.007; Fig. S1A), underscoring the clinical relevance of the PSMC5-METTL14 axis. To validate our *in silico* findings, we next enrolled 146 CRC patients to generate an in-house cohort. Expression levels of PSMC5 and METTL14 were tested by IHC. PSMC5 and METTL14 exhibited a reciprocal expression pattern correlated with tumor progression and patient survival (Fig. 2A-D). PSMC5 increased with advanced tumor stage (Fig. 2G), lymph node metastasis (N0 to N2) (Fig. 2F), distant metastasis (M1) (Fig. 2E), and served as an independent risk factor for poor overall survival (Fig. 2I; Table S1). Conversely, METTL14 expression decreased with advancing T stage (Fig. S2A), nodal involvement (Fig. S2B), overall stage (Fig. 2H), and was identified as an independent protective factor (Fig. 2J; Table S2; Fig. S2E). Moreover, stratification of the institutional cohort by combined PSMC5 and METTL14 immunohistochemical expression similarly revealed significantly different survival outcomes among the four groups, further supporting the prognostic relevance of the PSMC5-METTL14 axis in CRC (Fig. S2F). Together, these data define a PSMC5-METTL14 regulatory axis in CRC and link their expression directly to lymph-node and distant spread.

### PSMC5-driven proliferative and EMT phenotypes in CRC cells could be reversed by METTL14

**Figure 3:**
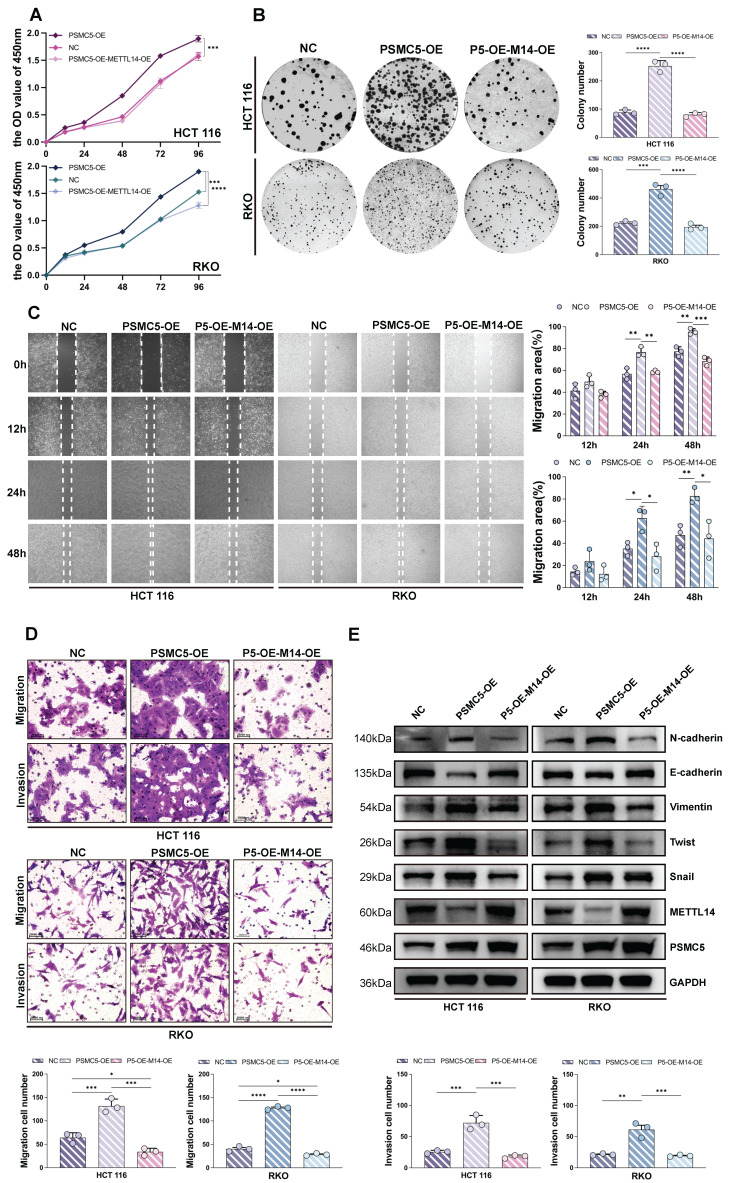
** PSMC5 drives CRC cell proliferation and motility while METTL14 attenuates these effects. (A)** CCK-8 growth curves (OD450, 0-96 h) of HCT116 and RKO cells under three conditions: negative control (NC), PSMC5 overexpression (PSMC5-OE), and PSMC5-OE plus METTL14 overexpression (P5-OE-M14-OE). **(B)** Representative colony-formation images and quantification (colonies/well) for the same groups in HCT116 and RKO. **(C)** Wound-healing assays at 0, 12, 24 and 48h with percent migration area quantified. **(D)** Transwell migration (no Matrigel) and invasion (Matrigel) assays with representative fields and cell counts per field. **(E)** Immunoblotting of EMT-related proteins (N-cadherin, E-cadherin, Vimentin, Twist, Snail) together with METTL14 and PSMC5; GAPDH as loading control. Data are presented as mean ± SD from three independent biological replicates. Statistical analysis: two-way ANOVA with Tukey's multiple-comparison test for (A, C); one-way ANOVA with Tukey's multiple-comparison test for (B, D). *P<0.05, **P<0.01, ***P<0.001, ****P<0.0001, ns, not significant.

To investigate the biological function of the PSMC5-METTL14 axis, we engineered HCT116 and RKO cells to overexpress PSMC5 either alone or in combination with METTL14. CCK-8 assays revealed that PSMC5-overexpressing (PSMC5-OE) cells grew significantly faster than the control group. Notably, co-expression of METTL14 markedly attenuated this accelerated growth in both cell lines (Fig. 3A). Similarly, colony formation ability was enhanced by PSMC5 overexpression but significantly suppressed upon METTL14 reintroduction (Fig. 3B). Wound-healing assays demonstrated more rapid gap closure in PSMC5-OE cells between 12-48 h, whereas METTL14 co-expression slowed the closure rate (Fig. 3C). In line with these results, transwell assays indicated that PSMC5-OE enhanced both migration and invasion abilities in both cell types, and these effects were counteracted by METTL14 co-expression, as evidenced by reduced numbers of migrating and invading cells (Fig. 3D). Furthermore, PSMC5 overexpression promoted EMT, with increased N-cadherin, Vimentin, Twist, and Snail and decreased E-cadherin and METTL14 at the protein level, accompanied by concordant mRNA changes in both HCT116 and RKO cells (Fig. 3E, Fig. S3A). In conclusion, these data suggest that PSMC5 enhances invasion, migration, proliferation, and EMT within *in vitro* colorectal cancer cells. At the mRNA level, PSMC5 overexpression also increased SOX4 expression (Fig. S3A). As SOX4 is a reported prometastatic target suppressed by METTL14-dependent m⁶A regulation in CRC, this result further supports that METTL14 functions downstream of PSMC5. Importantly, PSMC5 upregulation leads to reduced METTL14 expression, and restoring METTL14 levels can rescue these malignant phenotypes, supporting its role as a downstream effector in PSMC5-mediated oncogenic processes.

### PSMC5 couples SMURF1 to post-translational loss of METTL14

**Figure 4:**
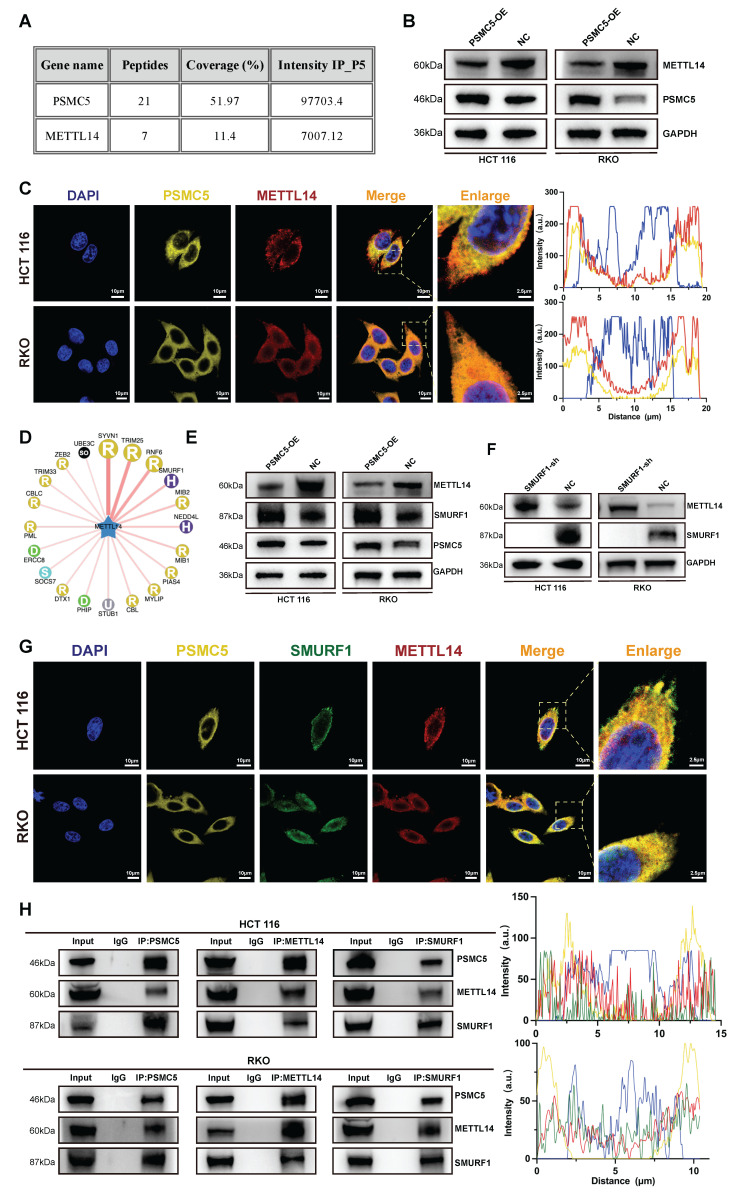
** PSMC5 forms a complex with METTL14 and SMURF1 and reduces METTL14 abundance. (A)** IP-MS of PSMC5 immunocomplexes: table lists identified proteins, peptide counts, sequence coverage, and IP intensities; METTL14 was detected, alongside PSMC5 bait. **(B)** Western blots of HCT116 and RKO cells showing METTL14 protein in control (NC) versus PSMC5-overexpressing (PSMC5-OE) cells; GAPDH, loading control. **(C)** Confocal IF for DAPI (blue), PSMC5 (yellow) and METTL14 (red) with merged/zoomed views; right, line-scan intensity profiles across indicated ROIs. Scale bars, 10 µm (main view) and 2.5 µm (inset). **(D)** Predicted E3 ligase network for METTL14 generated from UbBrowser, highlighting SMURF1 as a central candidate. **(E)** Western blots in NC vs PSMC5-OE cells for METTL14, SMURF1 and PSMC5. **(F)** Western blots showing METTL14 restoration upon SMURF1 knockdown (SMURF1-sh) in HCT116 and RKO. **(G)** Four-channel IF for DAPI (blue), PSMC5 (yellow), SMURF1 (green) and METTL14 (red) with merged/zoomed views. Scale bars, 10 µm (main view) and 2.5 µm (inset). Below, representative multi-channel line-scan profiles. **(H)** Reciprocal co-immunoprecipitation assays in HCT116 and RKO cells demonstrated that endogenous PSMC5, METTL14, and SMURF1 were present in the same immunoprecipitated complexes. Immunoprecipitation of PSMC5, METTL14, or SMURF1 followed by immunoblotting confirmed the associations between PSMC5 and METTL14, METTL14 and SMURF1, as well as PSMC5 and SMURF1 in both cell lines. IgG was used as a negative control.

To investigate the mechanism underlying PSMC5-induced METTL14 downregulation, we mapped the PSMC5 interactome. Immunoprecipitation-mass spectrometry (IP-MS) analysis of PSMC5 complexes identified METTL14 (7 peptides; ~11% sequence coverage), along with the bait protein PSMC5 (21 peptides; ~52% coverage), indicating a physical interaction between PSMC5 and METTL14 (Fig. 4A). Consistent with this, confocal microscopy revealed partial co-localization of PSMC5 and METTL14 in cytoplasmic and perinuclear regions, supported by overlapping fluorescence intensity profiles in line-scan analyses (Fig. 4C). Immunoblotting showed that PSMC5 overexpression markedly reduced METTL14 protein levels in HCT116 and RKO cells, whereas qRT-PCR revealed little change in METTL14 mRNA, supporting post-translational downregulation of METTL14 (Fig. 4B; Fig. S4A-B).

Since PSMC5 is a proteasome subunit, we hypothesized that PSMC5-mediated METTL14 downregulation occurs primarily via the ubiquitin-proteasome pathway. Given the central role of E3 ubiquitin ligases in substrate recognition and ubiquitination 38, we screened candidate E3 ligases targeting METTL14 using UbBrowser and identified SMURF1 as a top candidate (Fig. 4D). In HCT116 and RKO cells, PSMC5 overexpression reduced METTL14 expression while increasing SMURF1 expression at protein levels (Fig. 4E). SMURF1 knockdown restored METTL14 expression and suppressed xenograft growth, whereas concurrent METTL14 knockdown partially rescued this effect (Fig. 4F; Fig. S4I-M). Consistently, SMURF1 was elevated in our CRC cohort and was associated with advanced stage, metastasis, and worse overall survival (Fig. S4C-H; Table S3). These findings support SMURF1 as a candidate E3 ligase mediating post-translational downregulation of METTL14 in CRC cells.

In addition, triple-label immunofluorescence and reciprocal co-immunoprecipitation assays demonstrated co-localization and associations among PSMC5, SMURF1, and METTL14 in both HCT116 and RKO cells (Fig. 4G, H). SMURF1 was also detected in the PSMC5 IP-MS dataset. Together, these findings support a model in which PSMC5 upregulation promotes SMURF1 expression and is associated with SMURF1-mediated post-translational loss of METTL14 in CRC.

### PSMC5 promotes SMURF1-dependent ubiquitination of METTL14

**Figure 5:**
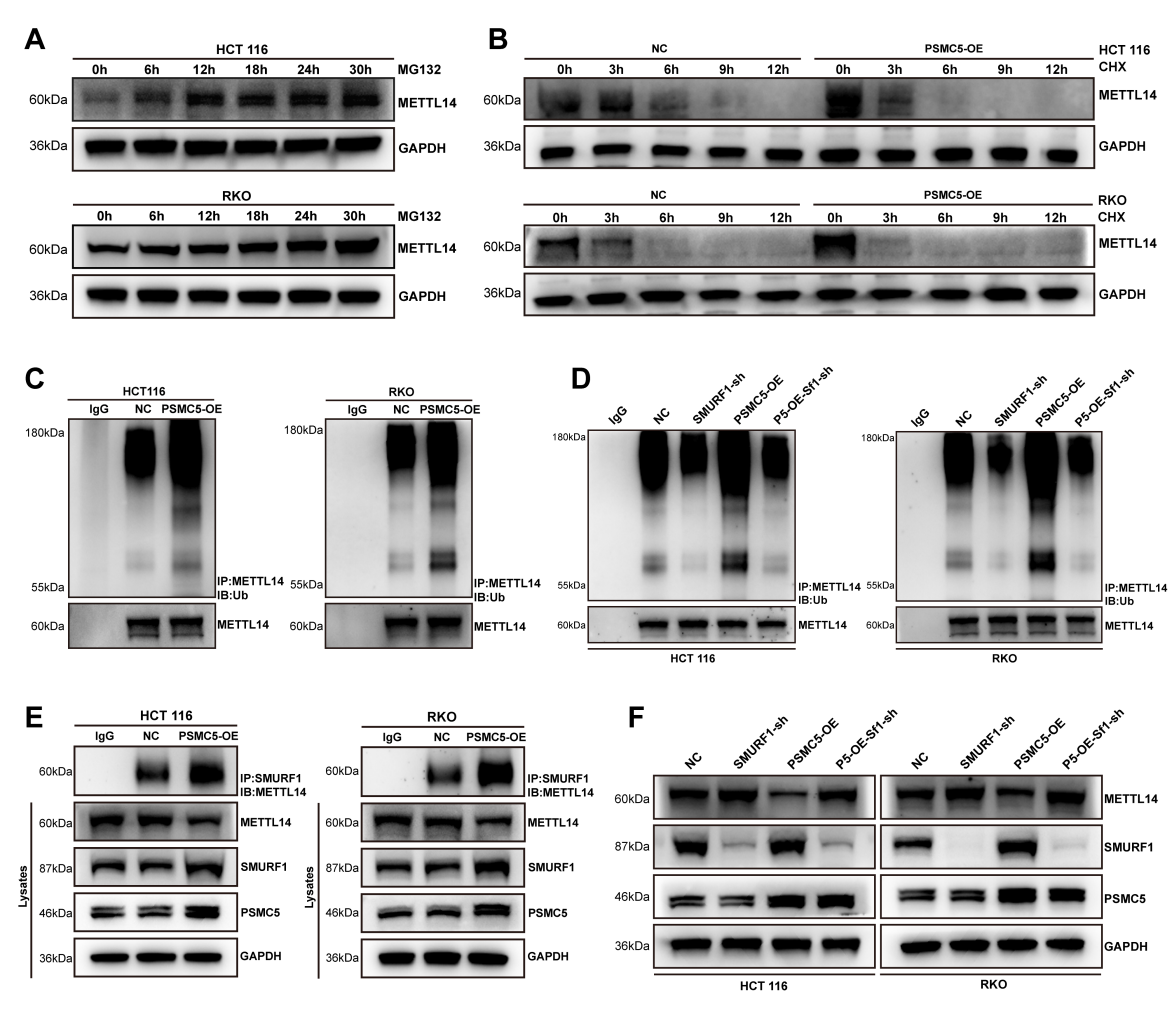
** PSMC5 promotes SMURF1-mediated ubiquitination of METTL14. (A)** Western blotting for METTL14 using HCT116 and RKO cell lines exposed to the proteasomal blocker MG132 (5 μM) at the specified time points. GAPDH was the loading control. **(B)** Western blotting for METTL14 in NC- and PSMC5-overexpressing HCT116 and RKO cells exposed to cycloheximide (CHX, 50 μg/mL) at the specified time points. GAPDH was the loading control. **(C)** METTL14 ubiquitination in control (NC) versus PSMC5-overexpressing (PSMC5-OE) cells. Cellular extracts underwent immunoprecipitation (IP) using anti-METTL14, followed by an immunoblot (IB) for ubiquitin; lower panels, IB-METTL14 in the IP. **(D)** Effect of SMURF1 depletion on METTL14 ubiquitination. Ubiquitin blots after IP-METTL14 in NC, SMURF1-sh, PSMC5-OE, and PSMC5-OE+SMURF1-sh conditions; corresponding IB-METTL14 in the IPs. **(E)** Co-immunoprecipitation of SMURF1 with METTL14 in NC and PSMC5-OE cells; Cellular extracts from RKO and HCT116 lines were subjected to immunoprecipitation employing an antibody targeting SMURF1. The resultant immunoprecipitates were subsequently probed via immunoblotting for METTL14. **(F)** Steady-state protein levels of METTL14, SMURF1, and PSMC5 in the same groups as in (C); GAPDH, loading control. Representative blots from ≥3 independent experiments.

To investigate the contribution of the ubiquitin-proteasome system to METTL14 loss caused by PSMC5, we inhibited the proteasome with MG132. This treatment caused a time-dependent accumulation of METTL14 in both cell lines, confirming proteasomal degradation (Fig. 5A). CHX chase assays further showed that PSMC5 overexpression accelerated METTL14 degradation in both cell lines, supporting a reduction in METTL14 protein stability (Fig. 5B). Subsequent ubiquitination assays revealed that PSMC5 overexpression intensified the poly-ubiquitin signal on METTL14 (Fig. 5C). Knocking down the E3 ligase SMURF1 significantly reduced both basal and PSMC5-enhanced METTL14 ubiquitination (Fig. 5D). Co-IP experiments also revealed that overexpressing PSMC5 enhanced the physical binding of SMURF1 with METTL14 (Fig. 5E). Consistent with this, PSMC5 upregulated SMURF1 and downregulated METTL14, while SMURF1 knockdown reversed the METTL14 loss induced by PSMC5 (Fig. 5F). Together, this evidence demonstrates that PSMC5 drives the SMURF1-mediated ubiquitination and subsequent proteasomal destruction of METTL14, explaining the antagonistic interplay between PSMC5 and METTL14 in CRC cells.

### PSMC5 promotes K11-linked ubiquitination of METTL14 at lysine 263

**Figure 6:**
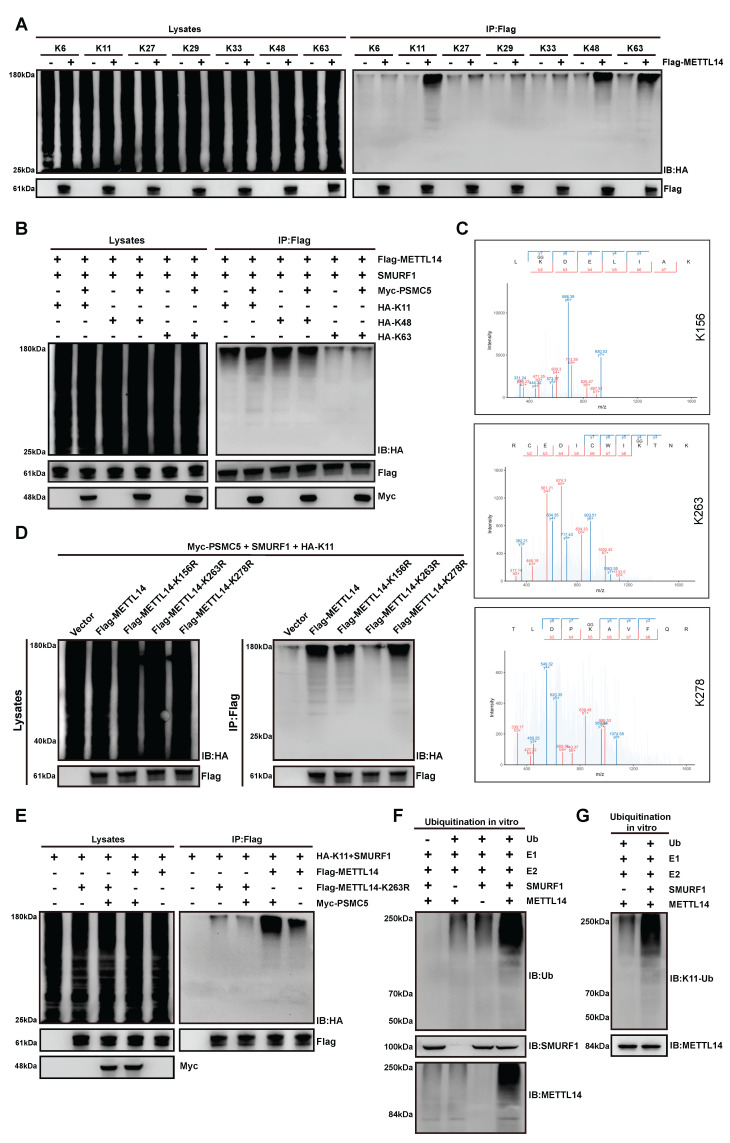
** PSMC5 promotes SMURF1-dependent K11-linked ubiquitination of METTL14 at K263. (A)** Ubiquitin linkage profiling of METTL14 in HEK293T cells using HA-tagged single-lysine ubiquitin mutants (K6, K11, K27, K29, K33, K48, and K63). Flag-METTL14 was immunoprecipitated and ubiquitination was detected by immunoblotting with anti-HA (right); whole-cell lysates are shown as input controls (left). **(B)** Effect of PSMC5 overexpression on METTL14 ubiquitination in the presence of HA-tagged K11-, K48-, or K63-only ubiquitin mutants in HEK293T cells. Flag-METTL14 was immunoprecipitated and ubiquitination was analyzed by anti-HA immunoblotting. **(C)** LC-MS/MS spectra identifying ubiquitinated lysine residues on METTL14, including K156, K263, and K278. **(D)** Site-directed mutagenesis of METTL14 (K156R, K263R, and K278R) in HEK293T cells co-expressing Myc-PSMC5 and HA-K11 ubiquitin. Flag-METTL14 was immunoprecipitated and probed with anti-HA. The K263R mutant markedly reduced K11-linked ubiquitination of METTL14. **(E)** Comparison of K11-linked ubiquitination between wild-type METTL14 and the K263R mutant in the presence or absence of PSMC5 overexpression in HEK293T cells. Flag-METTL14 was immunoprecipitated and ubiquitination was detected by anti-HA immunoblotting. **(F)** *In vitro* ubiquitination assay using purified proteins showing that SMURF1 directly catalyzed METTL14 ubiquitination. Ubiquitination was detected by anti-ubiquitin immunoblotting. **(G)** *In vitro* ubiquitination assay showing that SMURF1 mediated K11-linked ubiquitination of METTL14, detected by immunoblotting with an antibody against K11-linked ubiquitin chains. Unless otherwise indicated, HEK293T cells were co-transfected with SMURF1 together with the indicated constructs for *in vivo* ubiquitination assays. Representative blots are shown from three independent experiments.

We next sought to define the ubiquitin linkage preference and acceptor sites on METTL14 that mediate the destabilizing effect of PSMC5. Using HA-tagged single-lysine ubiquitin mutants in a reconstituted *in vivo* ubiquitination assay, we found that METTL14 underwent K11-, K48-, and K63-linked polyubiquitination (Fig. 6A). PSMC5 expression enhanced all three chain types, with the strongest effect observed on K11-linked ubiquitination (Fig. 6B). LC-MS/MS analysis identified K156, K263, and K278 as candidate ubiquitination sites on METTL14 (Fig. 6C). Functional validation with HA-K11 ubiquitin showed that the K263R mutation markedly impaired K11-linked ubiquitination of METTL14 in the presence of PSMC5, whereas K156R and K278R had relatively modest effects (Fig. 6D).

This result was further confirmed by direct comparison of wild-type METTL14 and the K263R mutant, which showed that PSMC5-induced K11-linked ubiquitination was substantially attenuated by K263 substitution (Fig. 6E). Consistent with this, re-expression of METTL14-K263R in PSMC5-overexpressing CRC cells markedly suppressed migration and invasion and partially reversed EMT-associated protein changes compared with wild-type METTL14 (Fig. S6A, B). Moreover, *in vitro* ubiquitination assays using purified proteins demonstrated that SMURF1 directly catalyzed METTL14 ubiquitination and mediated K11-linked ubiquitin chain formation on METTL14 (Fig. 6F, G). Collectively, these data indicate that PSMC5 promotes SMURF1-dependent K11-linked polyubiquitination of METTL14 primarily at K263, thereby accelerating its proteasomal degradation.

### SMURF1 knockdown attenuates PSMC5-driven tumor growth and metastasis *in vivo*

**Figure 7:**
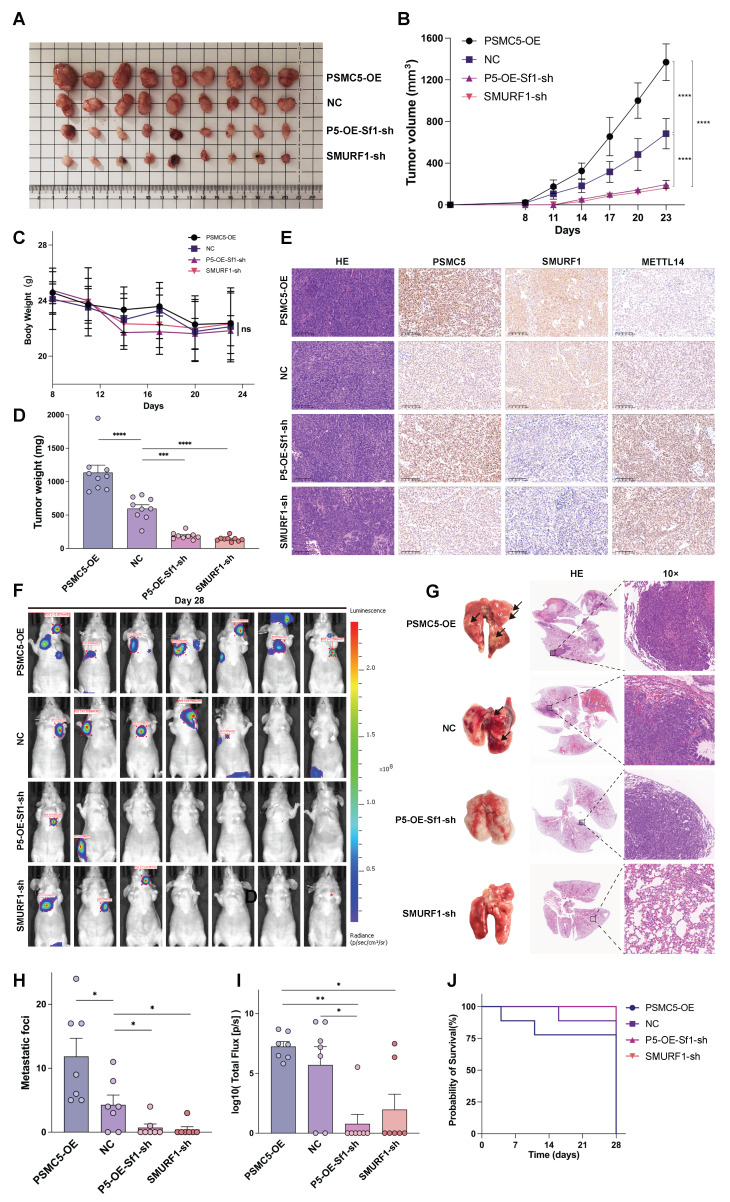
** PSMC5 enhances CRC progression *in vivo* and SMURF1 knockdown limits tumor growth and metastasis. (A)** Representative images of subcutaneous xenograft tumors excised from mice in the NC, PSMC5-OE, SMURF1-sh, or PSMC5-OE+SMURF1-sh groups. (n=9) **(B)** Tumor growth curves of subcutaneous xenografts formed by HCT116 cells with the indicated treatments. Tumor volumes were recorded at the indicated time points. **(C)** Body weight curves of mice in each group during the subcutaneous xenograft experiment. **(D)** Final tumor weights of subcutaneous xenografts at the endpoint of the experiment. **(E)** H&E staining, along with immunohistochemistry (IHC) targeting PSMC5, SMURF1, and METTL14 within the xenograft tumors (scale bar, 100 μm). **(F)** Typical bioluminescence images (n=9) showing pulmonary metastases at day 28, following tail vein administration of HCT116 cells in nude mice. **(G)** Typical images of whole lungs and H&E-stained lung sections. **(H, I)** Measurement of pulmonary metastatic nodules (H) and overall photon signal (I) for every group. **(J)** Kaplan-Meier survival analysis of mice (n=9). Data are presented as mean ± SD. Statistical analysis: two-way repeated-measures ANOVA with Tukey's multiple-comparison test for (B, C); one-way ANOVA with Tukey's multiple-comparison test for (D, H, I); log-rank test for (J). *P < 0.05, **P < 0.01, ***P < 0.001, ****P < 0.0001, ns, not significant.

We next assessed the *in vivo* significance of the PSMC5/SMURF1/METTL14 axis using a subcutaneous xenograft model. Representative xenograft tumors are shown in Fig. 7A. PSMC5 overexpression significantly accelerated tumor growth and increased final tumor weight compared with the control group (Fig. 7B, D). By contrast, SMURF1 knockdown inhibited xenograft growth and largely reversed the tumor-promoting effect of PSMC5 overexpression (Fig. 7A, B, D). No significant change in body weight was observed among the groups throughout the experiment (Fig. 7C). IHC analysis of tumor tissues confirmed the inverse relationship between PSMC5 and METTL14 *in vivo*. PSMC5-OE tumors exhibited high PSMC5 and low METTL14 expression, whereas SMURF1 knockdown restored METTL14 levels even in the presence of high PSMC5 (Fig. 7E).

In the lung metastasis model, PSMC5 overexpression strongly promoted metastatic dissemination, as shown by an increased number of mice with thoracic bioluminescence signals (Fig. 7F), more lung metastatic nodules, and higher total photon flux (Fig. 7G-I). Silencing SMURF1 significantly reduced metastasis in both control and PSMC5-OE backgrounds. Consistent with these findings, PSMC5-OE led to the shortest mouse survival, while SMURF1 knockdown extended survival irrespective of PSMC5 status (Fig. 7J). This *in vivo* evidence indicates that PSMC5 promotes tumor growth and metastasis, a process which relies upon SMURF1, and targeting this axis restores METTL14 expression and suppresses malignant progression.

### PSMC5-high epithelial states are associated with an immunosuppressive, myeloid-enriched spatial niche in colorectal cancer

**Figure 8:**
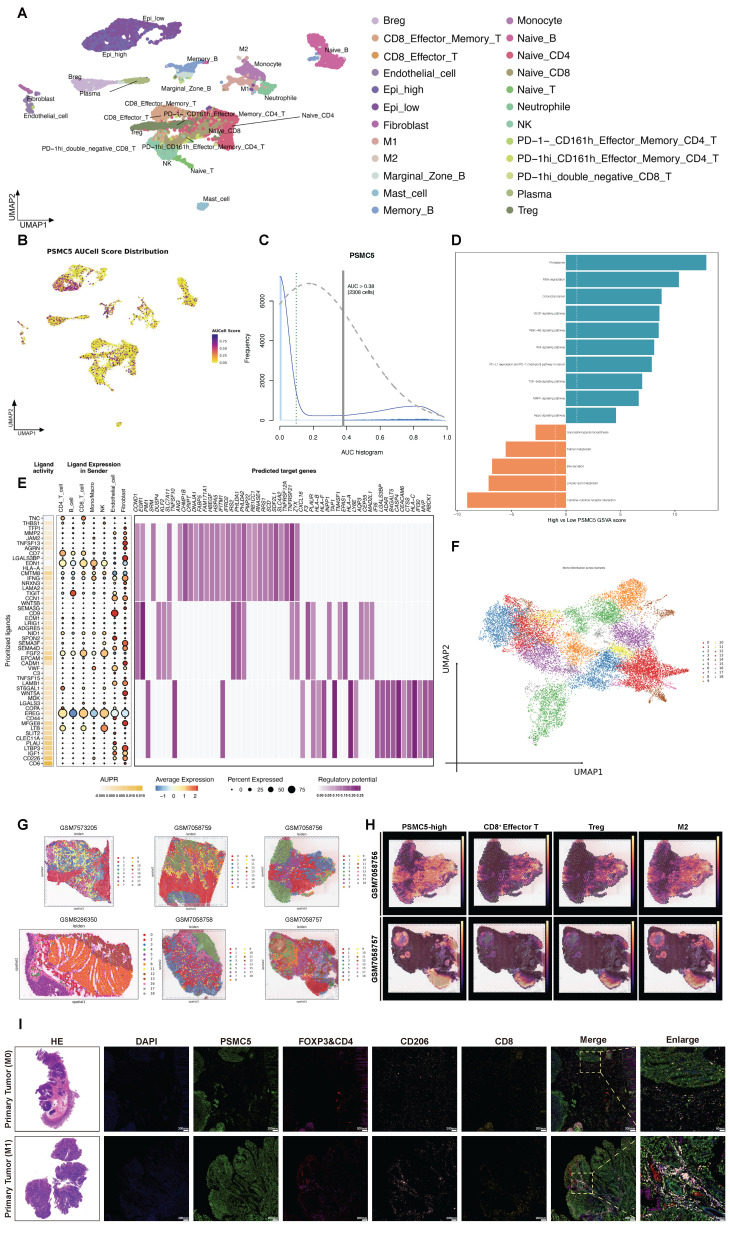
** Single-cell PSMC5 activity and spatial niches in colorectal cancer. (A)** UMAP visualization of annotated cell clusters, highlighting Epi_high and Epi_low subsets. **(B)** UMAP colored by PSMC5 AUCell score, showing enrichment in the Epi_high subset. **(C)** Distribution of PSMC5 AUCell scores; the vertical line marks the cutoff (AUC > 0.38) used to define PSMC5-high cells. **(D)** GSVA comparing PSMC5-high versus PSMC5-low epithelial cells. **(E)** Ligand-receptor-target matrix of prioritized PSMC5-associated ligands. Left, ligand activity (AUPR) and receptor connections, highlighting prominent ECM/adhesion ligands engaging integrin and growth-factor receptors. Right, regulatory potential on predicted target genes, converging on extracellular matrix remodeling, cell adhesion/migration and antigen-presentation/interferon-response programs. **(F)** UMAP embedding of all Visium spots from six sections grouped into transcriptionally defined niches. **(G)** Representative spatial transcriptomic maps from colorectal cancer and normal colonic tissue samples, including five colorectal cancer tumors (GSM7058756-GSM7058759; GSM7573205) and one normal colonic tissue (GSM8286350), showing Leiden-based niche assignment across the tissue sections. **(H)** Representative spatial distribution maps of PSMC5-high epithelial spots, CD8⁺ effector T cells, Tregs, and M2 macrophages in colorectal cancer samples. **(I)** Representative H&E staining and multiplex immunofluorescence images of formalin-fixed paraffin-embedded primary colorectal tumor sections from a stage I patient without metastasis and a stage IV patient with metastasis. Multiplex immunofluorescence staining was performed for PSMC5 (Alexa Fluor 488), CD206 (Cy7), FOXP3 (594), CD4 (Cy5), and CD8 (Cy3). Merged images and enlarged views are shown. Statistical analysis: Wilcoxon rank-sum test for (D).

To investigate the microenvironmental context associated with PSMC5, we analyzed single-cell RNA-seq data from four CRC samples in our cohort 39. After quality control, normalization, and clustering, 12,213 cells were resolved into 24 clusters representing major cell populations (Fig. 8A). Within the epithelial compartment, PSMC5 displayed a bimodal distribution, separating epithelial cells into PSMC5-high and PSMC5-low states on UMAP (Fig. 8B-C). GSVA showed that PSMC5-high epithelial cells were enriched for proteasome and RNA degradation pathways (Fig. 8D). Ligand-receptor analysis further identified a communication program enriched in ligands including TNC, THBS1, MMP2, WNT5A, and MDK, with downstream signatures related to extracellular matrix remodeling and antigen presentation (Fig. 8E, S7A). Differential expression analysis additionally linked the PSMC5-high epithelial state to EMT-related pathways, particularly TGF-β and Notch signaling (Fig. S8E).

To validate these findings spatially, we analyzed 10x Visium data from five CRC samples and one normal colonic tissue sample. Joint embedding and Leiden clustering identified 19 spatial states (Fig. 8F). Tumor tissues showed markedly greater architectural heterogeneity than normal tissues (Fig. 8G, S7B). In tumors, PSMC5-high epithelial regions were accompanied by enrichment of Tregs, M2 macrophages, fibroblasts, and CD8⁺ effector T cells, whereas such multicellular organization was not observed in normal tissues (Fig. S7C). Density mapping further showed that PSMC5-high epithelial spots were regionally associated with CD8⁺ effector T cells, Tregs, and M2 macrophages (Fig. 8H). Although direct spot-level overlap with immune subsets was limited (Fig. S8A-B), spatial association became more evident at the 200 μm paraspot scale, particularly for CD8⁺ effector/memory T cells, naïve T cells, and Tregs (Fig. S8C). Consistently, distance analysis showed that PSMC5-high epithelial spots were significantly closer to Treg and M2 macrophage spots than PSMC5-low epithelial spots in tumor tissues, whereas no such difference was observed in normal tissues (Fig. S8D). Differential interaction analysis further showed enhanced monocyte- and neutrophil-associated signaling, together with reduced fibroblast-M2 macrophage crosstalk (Fig. S8F).

We next performed multiplex immunofluorescence on primary tumors from stage I non-metastatic and stage IV metastatic CRC cases (Fig. 8I). Metastatic tumors exhibited higher PSMC5 expression, increased CD206⁺ M2-like macrophages and FOXP3⁺CD4⁺ Treg cells, and relatively fewer CD8⁺ T cells. Quantitative analysis confirmed increased densities of PSMC5⁺ and CD206⁺ cells, reduced CD8⁺ cells, and shorter distances from Treg and M2 cells to tumor nests in metastatic cases (Fig. S8G-I). Together, these results indicate that PSMC5-high epithelial states are associated with a spatially organized immune context characterized by Treg/M2 enrichment, relative CD8⁺ T-cell exclusion, and altered myeloid interactions.

## Discussion

Metastatic progression remains the principal determinant of poor outcome in CRC, with the 5-year survival rate dropping to below 15% once distant dissemination occurs 6. Although key signaling pathways and phenotypic programs involved in CRC metastasis have been extensively studied, the proteostatic mechanisms underlying this process remain incompletely defined. PSMC5, a core ATPase subunit of the 19S proteasomal regulatory particle, plays an important role in ubiquitin-dependent protein homeostasis and has shown prognostic significance across multiple human malignancies 40-42. Previous studies have linked PSMC5 to microglia-mediated inflammatory networks 43, neurodevelopmental abnormalities 44, CRC risk-related metabolic interactions 45, and TWIST-driven EMT 21. However, its role in metastatic progression has remained unclear.

Here, our study addresses this gap by identifying a PSMC5-SMURF1 axis that destabilizes METTL14 and links proteasomal regulation to epitranscriptomic control in CRC. Analyses of TCGA datasets and our institutional cohort consistently showed that PSMC5 is upregulated in CRC, particularly in tumors with lymph node or distant metastasis, and independently predicts unfavorable survival. Functional assays further demonstrated that PSMC5 enhances malignant phenotypes of CRC cells *in vitro* and *in vivo*, including proliferation, migration, invasion, tumor growth, and metastatic colonization. Together, these findings support PSMC5 not merely as a prognostic correlate, but as an active regulator of metastatic progression in CRC.

We next sought to determine how PSMC5 exerts these effects. Accumulating evidence indicates that METTL14 is regulated by the UPS. In human embryonic stem cells, the METTL3-METTL14 complex functions as a CRL4 adaptor, helping to degrade SUV39H1/H2 46, while in acute myeloid leukemia, METTL14 itself is degraded via the UPS upon WD6305 treatment 47. In bladder cancer, METTL14 promotes the stabilization of the USP38 transcript by applying m⁶A modification, a process that is contingent upon YTHDF2, and USP38 in turn acts as a deubiquitinase to stabilize METTL14 protein 48. Against this background, our study extends prior work by showing that METTL14 is not only an epitranscriptomic writer but also a metastasis-relevant proteostatic target in CRC. In this context, the PSMC5/METTL14 link is important because it connects proteasomal regulation to global m⁶A remodeling and places METTL14 downstream of a metastasis-promoting proteasomal axis. Together with the rescue effects of METTL14 re-expression, these findings support METTL14 as a key downstream effector through which PSMC5 translates post-translational regulation into epitranscriptomic and phenotypic reprogramming.

Importantly, this regulation also appears to be site-selective, as K263 emerged as the dominant acceptor site for PSMC5-enhanced ubiquitination of METTL14. The UPS regulates diverse cellular processes through distinct polyubiquitin chain linkages 49. Regarding the control of tumor metastasis, E3 ubiquitin ligases, together with deubiquitinating enzymes, are central components of the ubiquitination machinery 50, 51. Importantly, this linkage preference is mechanistically notable because K11-linked polyubiquitin chains, although less canonical than K48 chains, are established proteolytic signals, particularly in the turnover of cell-cycle regulators downstream of APC/C activity 52-54. In this context, our data suggest that METTL14 destabilization in CRC is not mediated predominantly through a conventional K48-dominant route, but instead involves a selective bias toward K11-linked ubiquitination. Given that K11-containing ubiquitin architectures have been linked to efficient proteasomal targeting and accelerated substrate turnover 54, 55, the preferential enhancement of K11-linked ubiquitination adds mechanistic depth to our model and suggests that PSMC5-SMURF1 regulation of METTL14 engages a non-canonical degradative ubiquitin code.

Our data further place SMURF1 as the major E3 node linking PSMC5 to METTL14 destabilization in CRC. This contrasts with the reported role of PSMC5 in promoting SMURF1 degradation in non-cancerous HEK293T cells 56. Given previous reports that CKIP-1 suppresses Smurf1 expression in colorectal cancer 57, we examined whether CKIP-1 restrains the PSMC5-SMURF1 branch. CKIP-1 was reduced in CRC cell lines relative to NCM460 cells, and its knockdown in PSMC5-overexpressing cells further increased SMURF1 expression and enhanced malignant phenotypes *in vitro* and *in vivo* (Fig. S5). These findings suggest that CKIP-1 may function as a negative regulator of the PSMC5-SMURF1 branch, consistent with its reported roles in suppressing SMURF1 translational synthesis 57 and facilitating SMURF1 self-degradation 56. Thus, CKIP-1 loss may amplify SMURF1 accumulation in PSMC5-high CRC.

*In vivo* results further support SMURF1 as a functional effector of the PSMC5/METTL14 axis in CRC, although the xenograft and experimental metastasis models used here do not fully recapitulate the complete course of spontaneous metastatic dissemination. This is consistent with recent evidence showing that, in KRAS-mutant CRC, SMURF1 promotes tumorigenesis by catalyzing PDK1 neddylation and activating PDK1-Akt signaling 58. In parallel, a PROTAC-based SMURF1 degrader has shown anti-tumor activity in colon cancer models 59, supporting the translational potential of SMURF1-targeted intervention. Our clinical cohort further showed that SMURF1 was elevated in CRC tissues and correlated with advanced stage, metastasis, and worse overall survival, reinforcing its clinical relevance in CRC progression. However, SMURF1 is broadly expressed and participates in normal physiological processes, including BMP-related development and bone homeostasis 31, 60. These features suggest that systemic inhibition may carry on-target toxicity. Therefore, SMURF1 is better regarded at present as a mechanistically supported candidate target rather than a fully validated therapeutic endpoint in CRC.

Beyond cell-intrinsic effects, accumulating evidence highlights the pivotal role of the tumor microenvironment (TME) in clinical outcome 6, 36. Effective antitumor immunity depends on the coordinated activation of helper CD4⁺ and cytotoxic CD8⁺ T cells, whereas permissive microenvironments are often characterized by functional exhaustion of effector populations and enrichment of immunosuppressive components such as regulatory T cells (Tregs) 6, 7. Whether high PSMC5 expression is linked to such immune contexts has remained unclear. By integrating single-cell and spatial transcriptomic analyses, we found that PSMC5-high epithelial states were associated with a spatially rewired niche rather than a purely cell-intrinsic program. PSMC5-high epithelial and associated stromal cells showed enrichment of ECM-remodeling and immune-modulatory ligands and were located within T cell-rich regions in which Treg- and M2-enriched zones surrounded PSMC5-high nests, whereas CD8⁺ effector T cells were largely confined to peripheral areas. These spatial inferences were further supported by multiplex immunofluorescence in primary tumor specimens from our institutional cohort. Myeloid interaction patterns were also selectively altered, consistent with a myeloid-enriched but functionally unbalanced microenvironment. Notably, SMURF1 also canonically regulates TGF-β/BMP signaling through SMAD turnover 31, 60, which may plausibly contribute in parallel to the immune phenotype observed here. PSMC5-high tumors may represent an immune-infiltrated but functionally restrained state, although these spatial inferences are derived from a relatively limited cohort and remain primarily association-based. This interpretation warrants future validation in immunocompetent models and in the context of immunotherapy response, including anti-PD-1-based settings. Previous studies also suggest that PSMC5 may influence treatment response in other tumor contexts, including radiosensitivity in lung cancer and chemoresistance in osteosarcoma 61-63, further supporting the broader translational relevance of this pathway.

## Conclusions

CRC metastasis reflects coordinated disruption of multi-layer regulatory programs rather than isolated alteration of individual oncogenes or tumor suppressors 64. In recent years, the epitranscriptome has emerged as a major theme in oncology, regarded as the third layer of gene regulation alongside genetics and epigenetics 65, yet how RNA-modifying proteins themselves are regulated remains poorly understood 30. Our study identifies a mechanistic link between proteasomal regulation and the epitranscriptome in CRC. Through SMURF1-dependent K11-linked ubiquitination of METTL14 at K263, PSMC5 destabilizes a core m⁶A writer and rewires epitranscriptomic regulation. This axis promotes EMT and metastatic progression and supports the PSMC5/SMURF1/METTL14 pathway as a candidate therapeutic vulnerability in metastatic CRC.

## Supplementary Material

Supplementary figures and tables.

## Data Availability

Publicly available datasets used in this study were obtained from the Gene Expression Omnibus (GEO) and UCSC Xena, including GSE39582, TCGA-COAD, TCGA-READ, and spatial transcriptomic datasets GSE225857 and GSE236698. The spatial datasets included five colorectal cancer samples (GSM7058756-GSM7058759 and GSM7573205) and one normal colonic tissue sample (GSM8286350). Newly generated datasets from this study, including MeRIP-seq, RNA-seq, and single-cell RNA-seq data, are available from the corresponding author upon reasonable request.

## Abbreviations

CRC: colorectal cancer
mCRC: metastatic colorectal cancer
m⁶A: N⁶-methyladenosine
EMT: epithelial-mesenchymal transition
UPS: ubiquitin-proteasome system
TME: tumor microenvironment
GEO: Gene Expression Omnibus
TCGA: The Cancer Genome Atlas
COAD: colon adenocarcinoma
READ: rectum adenocarcinoma
LIHC: liver hepatocellular carcinoma
LUAD: lung adenocarcinoma
OS: overall survival
KEGG: Kyoto Encyclopedia of Genes and Genomes
GO: Gene Ontology
DEG/DEGs: differentially expressed gene(s)
FDR: false discovery rate
MeRIP-seq: methylated RNA immunoprecipitation sequencing
TMA: tissue microarray
IHC: immunohistochemistry
IF: immunofluorescence
qRT-PCR: quantitative real-time polymerase chain reaction
BSA: bovine serum albumin
FBS: fetal bovine serum
PBS: phosphate-buffered saline
H-score: histochemistry score
CCK-8: cell counting kit-8
OD: optical density
SDS-PAGE: sodium dodecyl sulfate-polyacrylamide gel electrophoresis
PVDF: polyvinylidene fluoride
DAB: 3,3′-diaminobenzidine
DAPI: 4′,6-diamidino-2-phenylindole
HRP: horseradish peroxidase
LC-MS/MS: liquid chromatography-tandem mass spectrometry
IP-MS: immunoprecipitation-mass spectrometry
Co-IP: co-immunoprecipitation
IgG: immunoglobulin G
CHX: cycloheximide
IVIS: *in vivo* imaging system
H&E: hematoxylin and eosin
UMAP: uniform manifold approximation and projection
GSVA: gene set variation analysis
ECM: extracellular matrix
PTM: post-translational modification
TSA: tyramide signal amplification
DIA: data-independent acquisition
SPF: specific pathogen-free
CMS1: consensus molecular subtype 1
MSI: microsatellite instability

## References

[1] Bray F, Laversanne M, Sung H, Ferlay J, Siegel RL, Soerjomataram I (2024). Global cancer statistics 2022: GLOBOCAN estimates of incidence and mortality worldwide for 36 cancers in 185 countries. CA Cancer J Clin.

[2] Burnett-Hartman AN, Lee JK, Demb J, Gupta S (2021). An Update on the Epidemiology, Molecular Characterization, Diagnosis, and Screening Strategies for Early-Onset Colorectal Cancer. Gastroenterology.

[3] Eng C, Yoshino T, Ruíz-García E, Mostafa N, Cann CG, O'Brian B (2024). Colorectal cancer. Lancet.

[4] Ciardiello F, Ciardiello D, Martini G, Napolitano S, Tabernero J, Cervantes A (2022). Clinical management of metastatic colorectal cancer in the era of precision medicine. CA Cancer J Clin.

[5] Biller LH, Schrag D (2021). Diagnosis and Treatment of Metastatic Colorectal Cancer: A Review. Jama.

[6] Cañellas-Socias A, Sancho E, Batlle E (2024). Mechanisms of metastatic colorectal cancer. Nat Rev Gastroenterol Hepatol.

[7] Shin AE, Giancotti FG, Rustgi AK (2023). Metastatic colorectal cancer: mechanisms and emerging therapeutics. Trends Pharmacol Sci.

[8] Aiello NM, Maddipati R, Norgard RJ, Balli D, Li J, Yuan S (2018). EMT Subtype Influences Epithelial Plasticity and Mode of Cell Migration. Dev Cell.

[9] Zhang N, Ng AS, Cai S, Li Q, Yang L, Kerr D (2021). Novel therapeutic strategies: targeting epithelial-mesenchymal transition in colorectal cancer. Lancet Oncol.

[10] Bakir B, Chiarella AM, Pitarresi JR, Rustgi AK (2020). EMT, MET, Plasticity, and Tumor Metastasis. Trends Cell Biol.

[11] Liu F, Chen J, Li K, Li H, Zhu Y, Zhai Y (2024). Ubiquitination and deubiquitination in cancer: from mechanisms to novel therapeutic approaches. Mol Cancer.

[12] Aliabadi F, Sohrabi B, Mostafavi E, Pazoki-Toroudi H, Webster TJ (2021). Ubiquitin-proteasome system and the role of its inhibitors in cancer therapy. Open Biol.

[13] Cockram PE, Kist M, Prakash S, Chen SH, Wertz IE, Vucic D (2021). Ubiquitination in the regulation of inflammatory cell death and cancer. Cell Death Differ.

[14] Cui Z, Cong M, Yin S, Li Y, Ye Y, Liu X (2024). Role of protein degradation systems in colorectal cancer. Cell Death Discov.

[15] Sahu I, Glickman MH (2021). Proteasome in action: substrate degradation by the 26S proteasome. Biochem Soc Trans.

[16] Bard JAM, Goodall EA, Greene ER, Jonsson E, Dong KC, Martin A (2018). Structure and Function of the 26S Proteasome. Annu Rev Biochem.

[17] Marquez-Lona EM, Torres-Machorro AL, Gonzales FR, Pillus L, Patrick GN (2017). Phosphorylation of the 19S regulatory particle ATPase subunit, Rpt6, modifies susceptibility to proteotoxic stress and protein aggregation. PLoS One.

[18] Palve V, Pareek M, Krishnan NM, Siddappa G, Suresh A, Kuriakose MA (2018). A minimal set of internal control genes for gene expression studies in head and neck squamous cell carcinoma. PeerJ.

[19] Jhaveri DT, Kim MS, Thompson ED, Huang L, Sharma R, Klein AP (2016). Using Quantitative Seroproteomics to Identify Antibody Biomarkers in Pancreatic Cancer. Cancer Immunol Res.

[20] Kao TJ, Wu CC, Phan NN, Liu YH, Ta HDK, Anuraga G (2021). Prognoses and genomic analyses of proteasome 26S subunit, ATPase (PSMC) family genes in clinical breast cancer. Aging (Albany NY).

[21] He Z, Yang X, Huang L, Zhou L, Zhang S, Sun J (2021). PSMC5 Promotes Proliferation and Metastasis of Colorectal Cancer by Activating Epithelial-Mesenchymal Transition Signaling and Modulating Immune Infiltrating Cells. Front Cell Dev Biol.

[22] Jiang X, Liu B, Nie Z, Duan L, Xiong Q, Jin Z (2021). The role of m6A modification in the biological functions and diseases. Signal Transduct Target Ther.

[23] An Y, Duan H (2022). The role of m6A RNA methylation in cancer metabolism. Mol Cancer.

[24] Dou X, Huang L, Xiao Y, Liu C, Li Y, Zhang X (2023). METTL14 is a chromatin regulator independent of its RNA N6-methyladenosine methyltransferase activity. Protein Cell.

[25] Zhang C, Chen L, Liu Y, Huang J, Liu A, Xu Y (2021). Downregulated METTL14 accumulates BPTF that reinforces super-enhancers and distal lung metastasis via glycolytic reprogramming in renal cell carcinoma. Theranostics.

[26] Liu J, Eckert MA, Harada BT, Liu SM, Lu Z, Yu K (2018). m(6)A mRNA methylation regulates AKT activity to promote the proliferation and tumorigenicity of endometrial cancer. Nat Cell Biol.

[27] Ma JZ, Yang F, Zhou CC, Liu F, Yuan JH, Wang F (2017). METTL14 suppresses the metastatic potential of hepatocellular carcinoma by modulating N(6) -methyladenosine-dependent primary MicroRNA processing. Hepatology.

[28] Yang X, Zhang S, He C, Xue P, Zhang L, He Z (2020). METTL14 suppresses proliferation and metastasis of colorectal cancer by down-regulating oncogenic long non-coding RNA XIST. Mol Cancer.

[29] Chen X, Xu M, Xu X, Zeng K, Liu X, Pan B (2020). METTL14-mediated N6-methyladenosine modification of SOX4 mRNA inhibits tumor metastasis in colorectal cancer. Mol Cancer.

[30] Wang Y, Wang Y, Patel H, Chen J, Wang J, Chen ZS (2023). Epigenetic modification of m(6)A regulator proteins in cancer. Mol Cancer.

[31] Zhu H, Kavsak P, Abdollah S, Wrana JL, Thomsen GH (1999). A SMAD ubiquitin ligase targets the BMP pathway and affects embryonic pattern formation. Nature.

[32] Xia Q, Li Y, Han D, Dong L (2021). SMURF1, a promoter of tumor cell progression?. Cancer Gene Ther.

[33] Cao Y, Zhang L (2013). A Smurf1 tale: function and regulation of an ubiquitin ligase in multiple cellular networks. Cell Mol Life Sci.

[34] Liu J, Chen Y, Huang Q, Liu W, Ji X, Hu F (2018). IRAK2 counterbalances oncogenic Smurf1 in colon cancer cells by dictating ER stress. Cell Signal.

[35] Xia Q, Zhang H, Zhang P, Li Y, Xu M, Li X (2020). Oncogenic Smurf1 promotes PTEN wild-type glioblastoma growth by mediating PTEN ubiquitylation. Oncogene.

[36] Hanahan D (2022). Hallmarks of Cancer: New Dimensions. Cancer Discov.

[37] Zhan T, Faehling V, Rauscher B, Betge J, Ebert MP, Boutros M (2021). Multi-omics integration identifies a selective vulnerability of colorectal cancer subtypes to YM155. Int J Cancer.

[38] Wang T, Wang W, Wang Q, Xie R, Landay A, Chen D (2020). The E3 ubiquitin ligase CHIP in normal cell function and in disease conditions. Ann N Y Acad Sci.

[39] Xu X, Liu J, Qin W, Ding C, Cai Z, Shu D (2025). ST6GAL1-Mediated Sialylation Stabilizes PD-L1 and Drives Immunosuppressive Tumor Microenvironment in Colorectal Cancer. Adv Sci (Weinh).

[40] Yu ZQ, Carmichael J, Collins GA, D'Agostino MD, Lessard M, Firth HV (2024). PSMC5 insufficiency and P320R mutation impair proteasome function. Hum Mol Genet.

[41] Tychhon B, Allen JC, Gonzalez MA, Olivas IM, Solecki JP, Keivan M (2023). The prognostic value of 19S ATPase proteasome subunits in acute myeloid leukemia and other forms of cancer. Front Med (Lausanne).

[42] Spano D, Catara G (2023). Targeting the Ubiquitin-Proteasome System and Recent Advances in Cancer Therapy. Cells.

[43] Bi W, Bao K, Zhou X, Deng Y, Li X, Zhang J (2023). PSMC5 regulates microglial polarization and activation in LPS-induced cognitive deficits and motor impairments by interacting with TLR4. J Neuroinflammation.

[44] Vinci M, Musumeci A, Papa C, Ragalmuto A, Saccone S, Federico C (2025). Strengthening the Role of PSMC5 as a Potential Gene Associated with Neurodevelopmental Disorders. Int J Mol Sci.

[45] Xia Z, Su YR, Petersen P, Qi L, Kim AE, Figueiredo JC (2020). Functional informed genome-wide interaction analysis of body mass index, diabetes and colorectal cancer risk. Cancer Med.

[46] Shan Y, Zhang Y, Wei Y, Zhang C, Lin H, He J (2024). METTL3/METTL14 maintain human nucleoli integrity by mediating SUV39H1/H2 degradation. Nat Commun.

[47] Du W, Huang Y, Chen X, Deng Y, Sun Y, Yang H (2024). Discovery of a PROTAC degrader for METTL3-METTL14 complex. Cell Chem Biol.

[48] Huang J, Zhou W, Hao C, He Q, Tu X (2022). The feedback loop of METTL14 and USP38 regulates cell migration, invasion and EMT as well as metastasis in bladder cancer. PLoS Genet.

[49] Husnjak K, Dikic I (2012). Ubiquitin-binding proteins: decoders of ubiquitin-mediated cellular functions. Annu Rev Biochem.

[50] Han S, Wang R, Zhang Y, Li X, Gan Y, Gao F (2022). The role of ubiquitination and deubiquitination in tumor invasion and metastasis. Int J Biol Sci.

[51] Ren Y, Chen D, Zhai Z, Chen J, Li A, Liang Y (2021). JAC1 suppresses proliferation of breast cancer through the JWA/p38/SMURF1/HER2 signaling. Cell Death Discov.

[52] Wickliffe KE, Williamson A, Meyer HJ, Kelly A, Rape M (2011). K11-linked ubiquitin chains as novel regulators of cell division. Trends Cell Biol.

[53] Matsumoto ML, Wickliffe KE, Dong KC, Yu C, Bosanac I, Bustos D (2010). K11-linked polyubiquitination in cell cycle control revealed by a K11 linkage-specific antibody. Mol Cell.

[54] Wu T, Merbl Y, Huo Y, Gallop JL, Tzur A, Kirschner MW (2010). UBE2S drives elongation of K11-linked ubiquitin chains by the anaphase-promoting complex. Proc Natl Acad Sci U S A.

[55] Meyer HJ, Rape M (2014). Enhanced protein degradation by branched ubiquitin chains. Cell.

[56] Wang Y, Nie J, Wang Y, Zhang L, Lu K, Xing G (2012). CKIP-1 couples Smurf1 ubiquitin ligase with Rpt6 subunit of proteasome to promote substrate degradation. EMBO Rep.

[57] Nie J, Liu L, Xing G, Zhang M, Wei R, Guo M (2014). CKIP-1 acts as a colonic tumor suppressor by repressing oncogenic Smurf1 synthesis and promoting Smurf1 autodegradation. Oncogene.

[58] Peng Z, Fang W, Wu B, He M, Li S, Wei J (2025). Targeting Smurf1 to block PDK1-Akt signaling in KRAS-mutated colorectal cancer. Nat Chem Biol.

[59] Xiong X, Zhao Y, Sun Y (2025). SMURF1: a promising target for colon cancer therapy. Protein Cell.

[60] Yamashita M, Ying SX, Zhang GM, Li C, Cheng SY, Deng CX (2005). Ubiquitin ligase Smurf1 controls osteoblast activity and bone homeostasis by targeting MEKK2 for degradation. Cell.

[61] Chen ZL, Xie C, Zeng W, Huang RQ, Yang JE, Liu JY (2024). Synergistic induction of mitotic pyroptosis and tumor remission by inhibiting proteasome and WEE family kinases. Signal Transduct Target Ther.

[62] Yim JH, Yun HS, Lee SJ, Baek JH, Lee CW, Song JY (2016). Radiosensitizing effect of PSMC5, a 19S proteasome ATPase, in H460 lung cancer cells. Biochem Biophys Res Commun.

[63] Pan Q, Yang W, Huang F, Wu W, Shao Z, Zhang Z (2025). EWSR1-PSMC5 fusion gene variously activating autophagy in drug resistance of osteosarcoma: A novel gene fusion model report and mechanism research. Genes Dis.

[64] Valastyan S, Weinberg RA (2011). Tumor metastasis: molecular insights and evolving paradigms. Cell.

[65] Orsolic I, Carrier A, Esteller M (2023). Genetic and epigenetic defects of the RNA modification machinery in cancer. Trends Genet.

