# Supplementary material for: PSMC5 Orchestrates an Immunosuppressive Niche and Metastasis in Colorectal Cancer via SMURF1-Mediated K11-Linked Ubiquitination of METTL14
Source: Int J Biol Sci. 2026 May 11;22(10):5182–202. doi: 10.7150/ijbs.130350 (PMC13215249; doi:10.7150/ijbs.130350)
Supplement: Supplementary file 1 — Supplementary figures and tables. [file ijbsv22p5182s1.pdf]

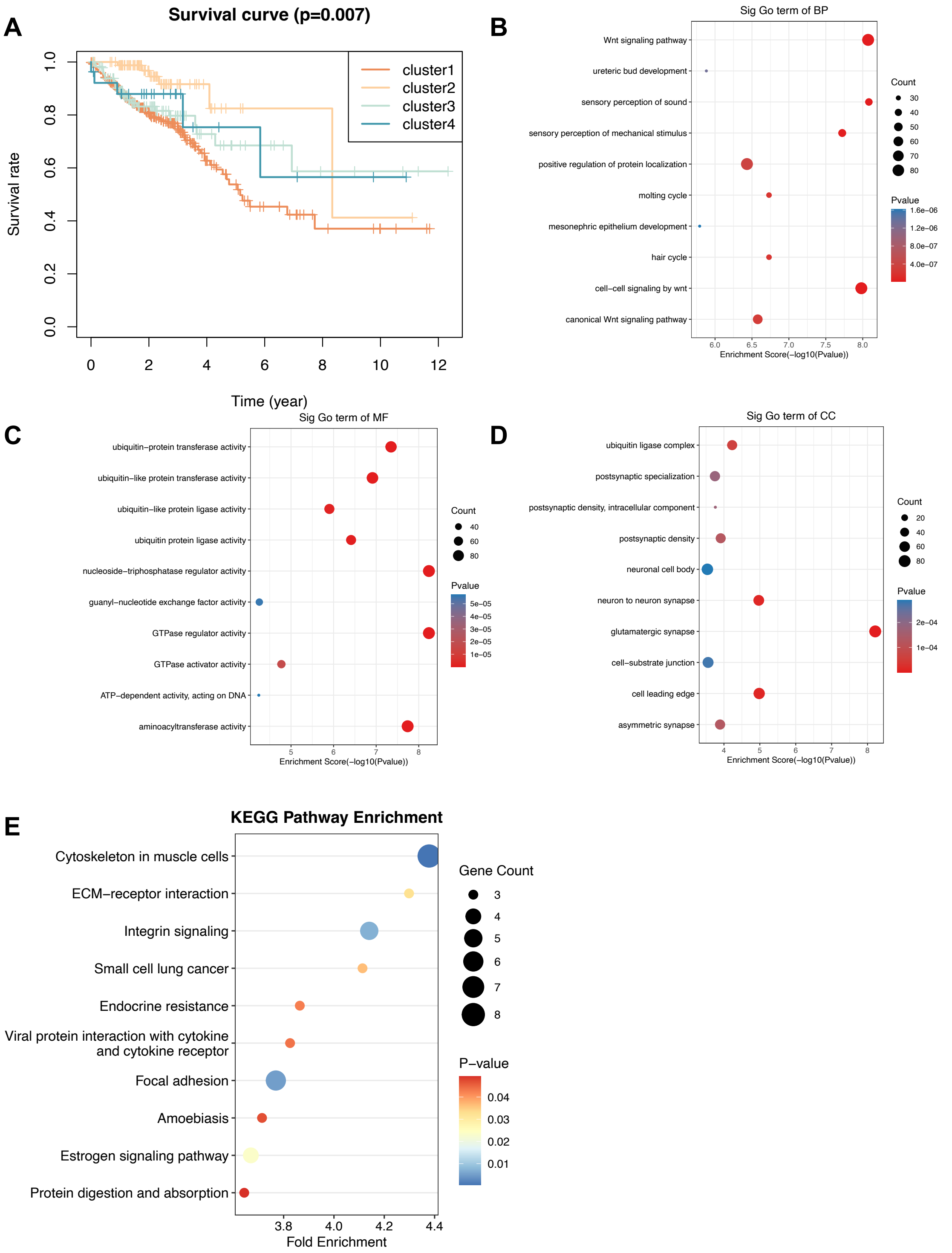

Figure S1 (A) Kaplan-Meier survival curves for TCGA colorectal cancer patients stratified into four groups by combined PSMC5 and METTL14 expression. Cluster 1: PSMC5 low and METTL14 low. Cluster 2: PSMC5 low and METTL14 high. Cluster 3: PSMC5 high and METTL14 low. Cluster 4: PSMC5 high and METTL14 high. (B) GO Biological Process (BP)

term enrichment for differentially methylated mRNAs resulting from PSMC5 overexpression. (C) GO Molecular Function (MF) term enrichment for differentially methylated mRNAs associated with PSMC5 overexpression. (D) GO Cellular Component (CC) term enrichment for differentially methylated mRNAs associated with PSMC5 overexpression. (E) KEGG pathway enrichment analysis of differentially expressed mRNAs between the PSMC5-overexpression and NC groups following RNA sequencing. Statistical analysis: log-rank test for (A); hypergeometric test for enrichment for (B-E).

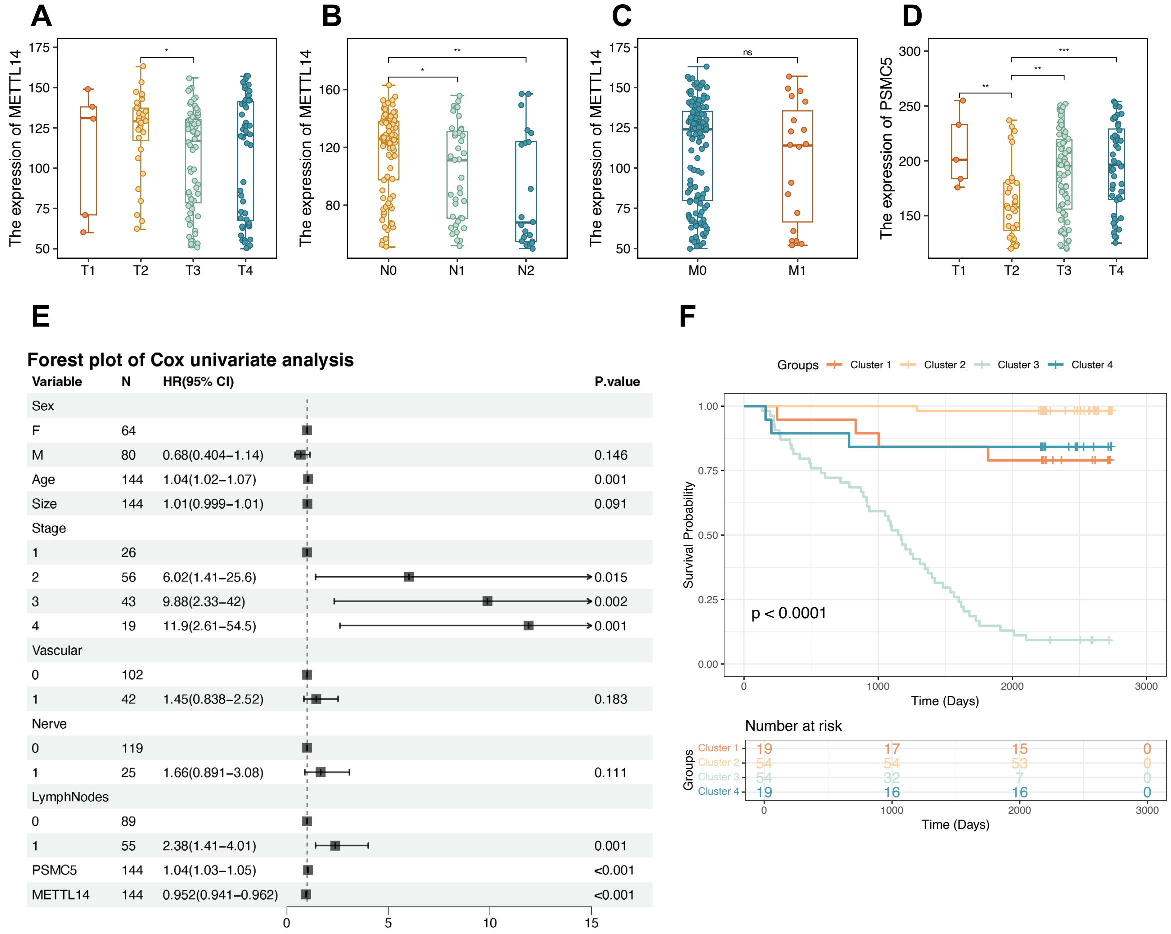

Figure S2 (A) METTL14 IHC scores stratified by T stage (T1-T4). (B) METTL14 IHC scores stratified by N stage (N0-N2). (C) METTL14 IHC scores stratified by M stage (M0/M1). (D) PSMC5 IHC scores stratified by T stage (T1-T4). (E) Univariate Cox analysis for overall survival including clinicopathologic variables and IHC scores of PSMC5 and METTL14; forest plot displays HRs and 95% CIs. Statistical tests as in Fig. 2. (F) Kaplan-Meier survival curves for the institutional cohort stratified by combined PSMC5 and METTL14 immunohistochemical expression into four groups: Cluster 1, PSMC5 low/METTL14 low; Cluster 2, PSMC5 low/METTL14 high; Cluster 3, PSMC5 high/METTL14 low; and Cluster 4, PSMC5 high/METTL14 high. The number of patients at risk is shown below. Statistical analysis: Kruskal-Wallis test with multiple-comparison correction for (A, B, D); Wilcoxon

rank-sum test for (C); Wald test from Cox proportional hazards regression for (E); log-rank test for (F). *P<0.05, **P<0.01, ***P<0.001, ****P<0.0001, ns, not significant.

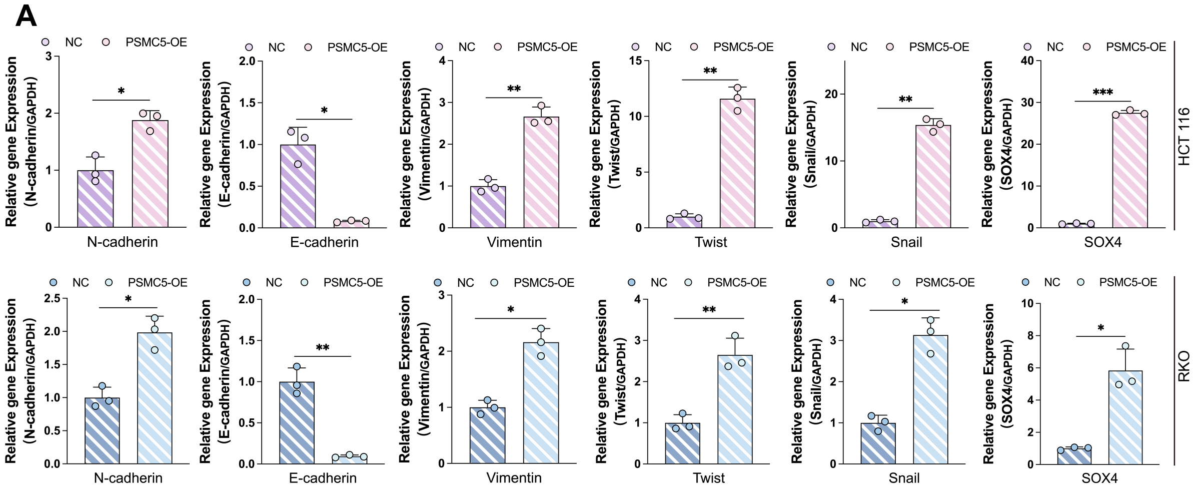

Figure S3 (A) qRT-PCR analysis of N-cadherin, E-cadherin, Vimentin, Twist, Snail, and SOX4 expression in HCT116 and RKO cells stably expressing control vector or PSMC5. Gene expression was normalized to GAPDH. Statistical analysis: unpaired two-tailed Student's t test. Data are presented as mean ± SD from three independent biological replicates. *P<0.05, **P<0.01, ***P<0.001.

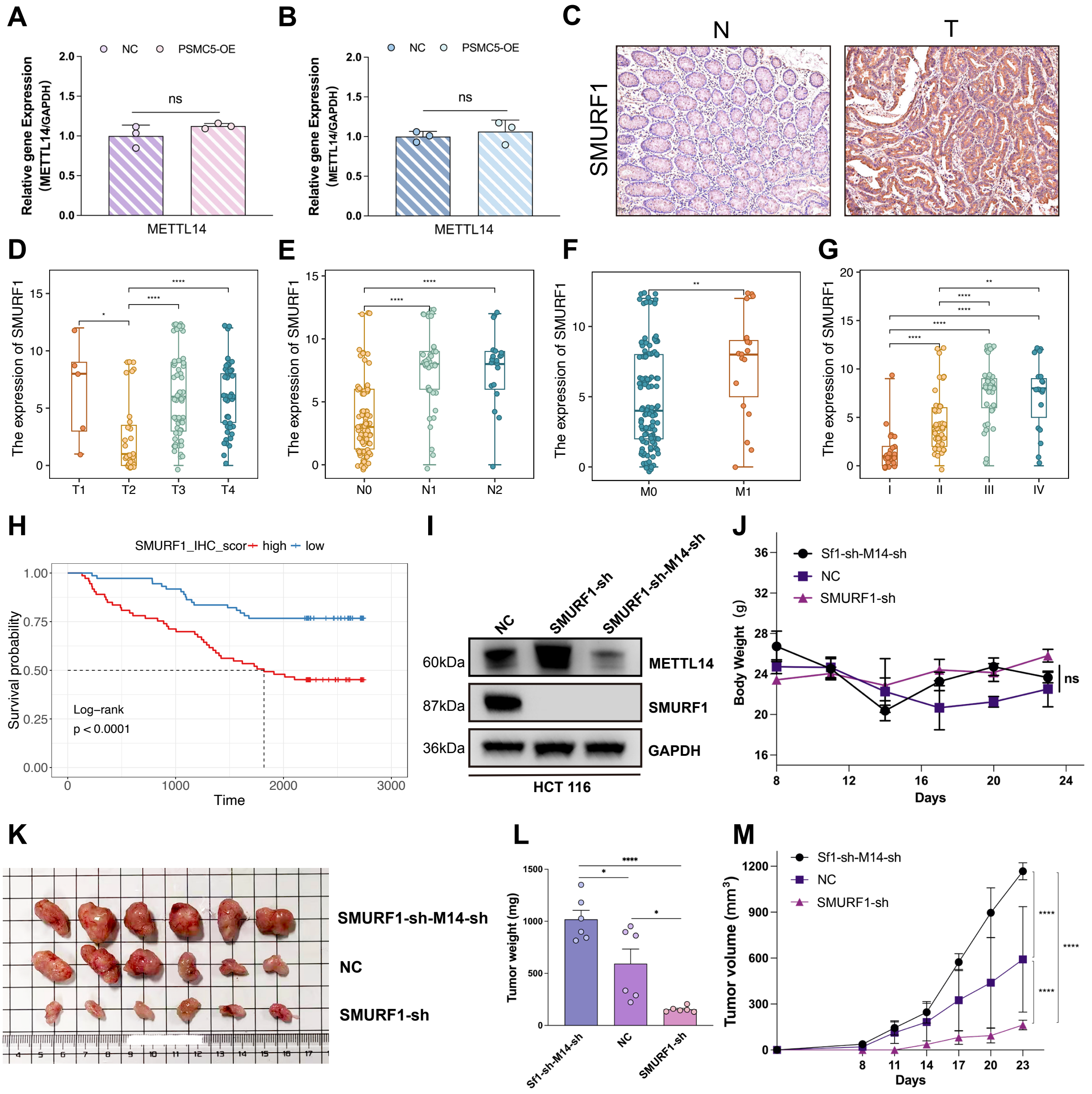

Figure S4 (A-B) qRT-PCR analysis of METTL14 mRNA expression in HCT116 and RKO cells with lentivirus-mediated stable overexpression of PSMC5 or corresponding control cells. Gene expression was normalized to GAPDH. (C) Representative immunohistochemical staining of SMURF1 in normal colorectal mucosa (N) and colorectal tumor tissue (T). (D-G) Association of SMURF1 expression with clinicopathological characteristics of colorectal cancer, including T stage (D), N stage (E), M stage (F), and pathological stage (G). (H) Kaplan-Meier overall survival curves for colorectal cancer patients stratified by SMURF1

immunohistochemical score (high vs low). (I) Immunoblotting of METTL14 and SMURF1 in HCT116 cells from the NC, SMURF1-sh, and SMURF1-sh+METTL14-sh groups. GAPDH served as a loading control. (J) Body weight curves of mice in the NC, SMURF1-sh, and SMURF1-sh+METTL14-sh groups during the subcutaneous xenograft experiment. (K) Representative images of subcutaneous xenograft tumors excised from mice in the NC, SMURF1-sh, and SMURF1-sh+METTL14-sh groups. (L) Final tumor weights of subcutaneous xenografts from mice in the NC, SMURF1-sh, and SMURF1-sh+METTL14-sh groups at the endpoint of the experiment. (M) Tumor growth curves of subcutaneous xenografts formed by HCT116 cells in the NC, SMURF1-sh, and SMURF1-sh+METTL14-sh groups. Tumor volumes were recorded at the indicated time points. Statistical analysis: unpaired two-tailed Student's t test for (A, B); Kruskal-Wallis test with multiple-comparison correction for (D, E, G); Wilcoxon rank-sum test for (F); log-rank test for (H); two-way repeated-measures ANOVA with multiple-comparison correction for (J, M); one-way ANOVA with multiple-comparison correction for (L). Panels (C, I, K) show representative images. *P<0.05, **P<0.01, ***P<0.001, ****P<0.0001, ns, not significant.

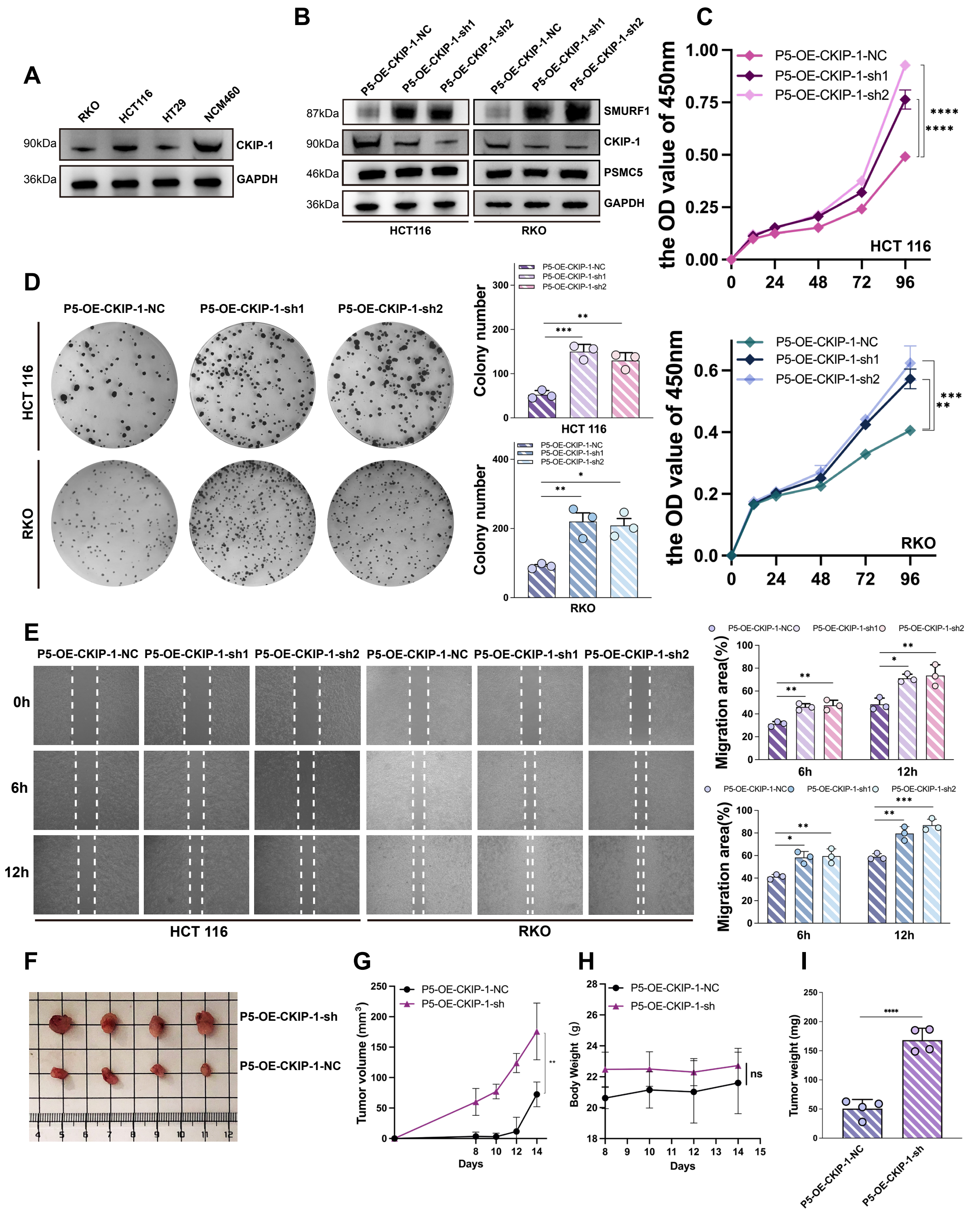

Figure S5 (A) Immunoblotting of CKIP-1 in RKO, HCT116, HT29, and NCM460 cells. GAPDH served as a loading control. (B) Immunoblotting of SMURF1, CKIP-1, and PSMC5 in HCT116 and RKO cells from the P5-OE-CKIP-1-NC, P5-OE-CKIP-1-sh1, and P5-OE-CKIP-1-sh2 groups. GAPDH served as a loading control. (C) CCK-8 growth curves of

HCT116 and RKO cells in the indicated groups. (D) Representative colony-formation images and quantification in HCT116 and RKO cells from the indicated groups. (E) Wound-healing assays with quantification of migration area in HCT116 and RKO cells from the indicated groups. (F) Representative images of subcutaneous xenograft tumors excised from nude mice in the P5-OE-CKIP-1-NC and P5-OE-CKIP-1-sh groups. (G) Tumor growth curves of subcutaneous xenografts in the indicated groups. (H) Body weight curves of mice in the indicated groups during the xenograft experiment. (I) Final tumor weights of subcutaneous xenografts at the experimental endpoint. Data are presented as mean ± SD. Statistical analysis: two-way repeated-measures ANOVA with Tukey's multiple-comparison test for (C, E, G, H); one-way ANOVA with Tukey's multiple-comparison test for (D); unpaired two-tailed Student's t test for (I). *P<0.05, **P<0.01, ***P<0.001, ****P<0.0001, ns, not significant.

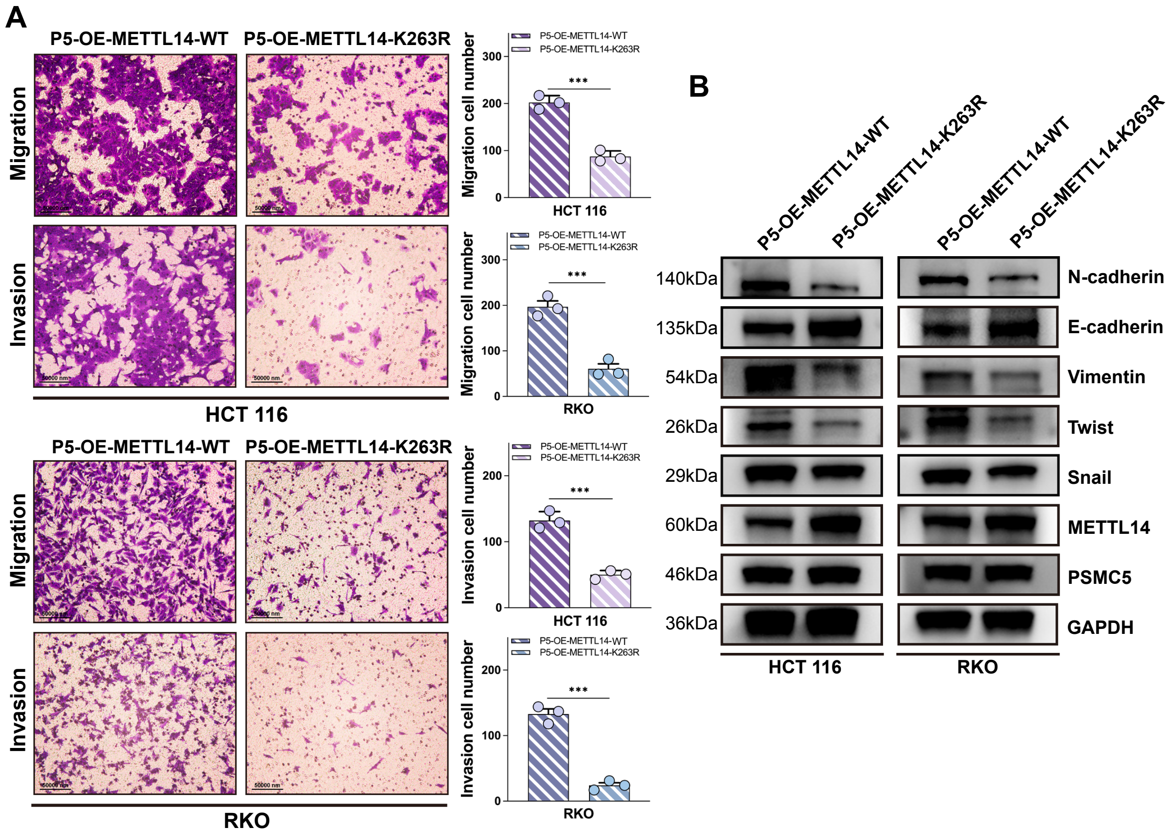

Figure S6 (A) Transwell migration (without Matrigel) and invasion (with Matrigel) assays with representative images and quantification of migrated or invaded cells in HCT116 and RKO cells with stable PSMC5 overexpression, followed by transfection with METTL14-WT or METTL14-K263R plasmids. (B) Immunoblotting of EMT-related proteins (N-cadherin, E-cadherin, Vimentin, Twist, and Snail) together with METTL14 and PSMC5 in HCT116 and RKO cells with stable PSMC5 overexpression, followed by transfection with METTL14-WT or METTL14-K263R plasmids. GAPDH served as a loading control. Data are presented as mean ± SD from three independent biological replicates. Statistical analysis: unpaired two-tailed Student's t test for the quantification in (A). Panel (B) shows representative immunoblot images. *P<0.05, **P<0.01, ***P<0.001.

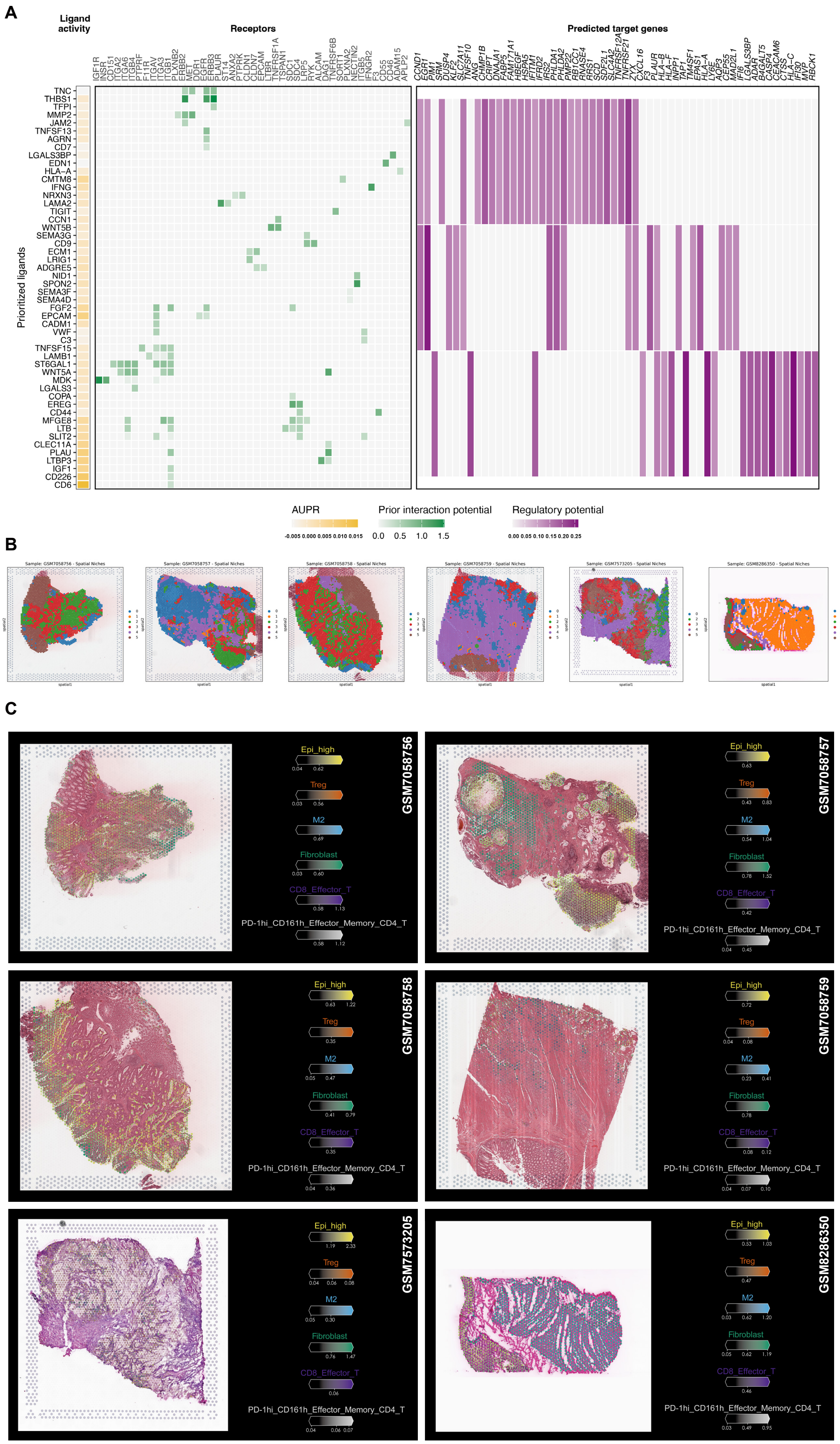

A
Ligand activity
Receptors
Predicted target genes
IGF1R INSR CD151 ITGA2 ITGA6 ITGB4 PTPRF F11R ITGAV ITGA3 ITGB1 PLXNB2 ERBB2 MET DDR1 EGFR ERBB3 PLAUR ST14 ANXA2 PTPRK CLDN1 CLDN7 EPCAM LTBR TNFRSF1A TSPAN1 SDC1 SDC4 LRP5 PTK7 ALCAM DAG1 TNFRSF6B SIRT1 PLXNA2 NECTIN2 ITGB5 IFNGR2 F3 CD55 CD46 ADAM15 APLP2
CCND1 EGR1 PIM1 SIK1 DUSP4 KLF2 SLC7A11 TNFSF10 ANG CHMP1B CRIPT DNAJA1 FABP5 FAM171A1 HBEGF HSPA5 IFITM1 IFRD2 IRS2 PHLDA1 PHLDA2 PMP22 RB1CC1 RNASE4 RRS1 SCD SDF2L1 SLC2A1 TNFRSF12A TNFRSF21 TXN CXCL16 F3 PLAUR HLA-B HLA-F INPP1 TAP1 TM4SF1 EPAS1 HLA-A LY6E AQP3 CEP55 MAD2L1 IFI6 LGALS3BP ADAR B4GALT5 CASP4 CEACAM6 CTSS HLA-C IFI30 MVP RBCK1
Prioritized ligands
TNC THBS1 TFPI MMP2 JAM2 TNFSF13 AGRN CD7 LGALS3BP EDN1 HLA-A CMTM8 IFNG NRXN3 LAMA2 TIGIT CCN1 WNT5B SEMA3G CD9 ECM1 LRIG1 ADGRE5 NID1 SPON2 SEMA3F SEMA4D FGF2 EPCAM CADM1 VWF C3 TNFSF15 LAMB1 ST6GAL1 WNT5A MDK LGALS3 COPA EREG CD44 MFGE8 LTB SLIT2 CLEC11A PLAU LTBP3 IGF1 CD226 CD6
AUPR
-0.005 0.000 0.005 0.010 0.015
Prior interaction potential
0.0 0.5 1.0 1.5
Regulatory potential
0.00 0.05 0.10 0.15 0.20 0.25
B
Sample: GSM7058756 - Spatial Niches
Sample: GSM7058757 - Spatial Niches
Sample: GSM7058758 - Spatial Niches
Sample: GSM7058759 - Spatial Niches
Sample: GSM7573205 - Spatial Niches
Sample: GSM8286350 - Spatial Niches
spatial1
spatial2
C
GSM7058756
GSM7058757
GSM7058758
GSM7058759
GSM7573205
GSM8286350
Epi_high
Treg
M2
Fibroblast
CD8_Effector_T
PD-1hi_CD161h_Effector_Memory_CD4_T

Figure S7 (A) Extended ligand-receptor-target landscape corresponding to Figure 8E, showing ligand activity (AUPR), receptor connectivity and regulatory potential on downstream target genes. (B) Spatial transcriptomic maps of six tissue sections, including five colorectal cancer tumors (GSM7058756-GSM7058759; GSM7573205) and one normal colonic tissue (GSM8286350), showing spatial niche assignment across the tissue sections. (C) Spatial distribution maps of selected cellular programs and immune/stromal components across the six tissue sections, including PSMC5-high epithelial cells, Tregs, M2 macrophages, fibroblasts, $CD8^+$ effector T cells, and PD-1-high-CD161-high effector memory $CD4^+$ T cells.

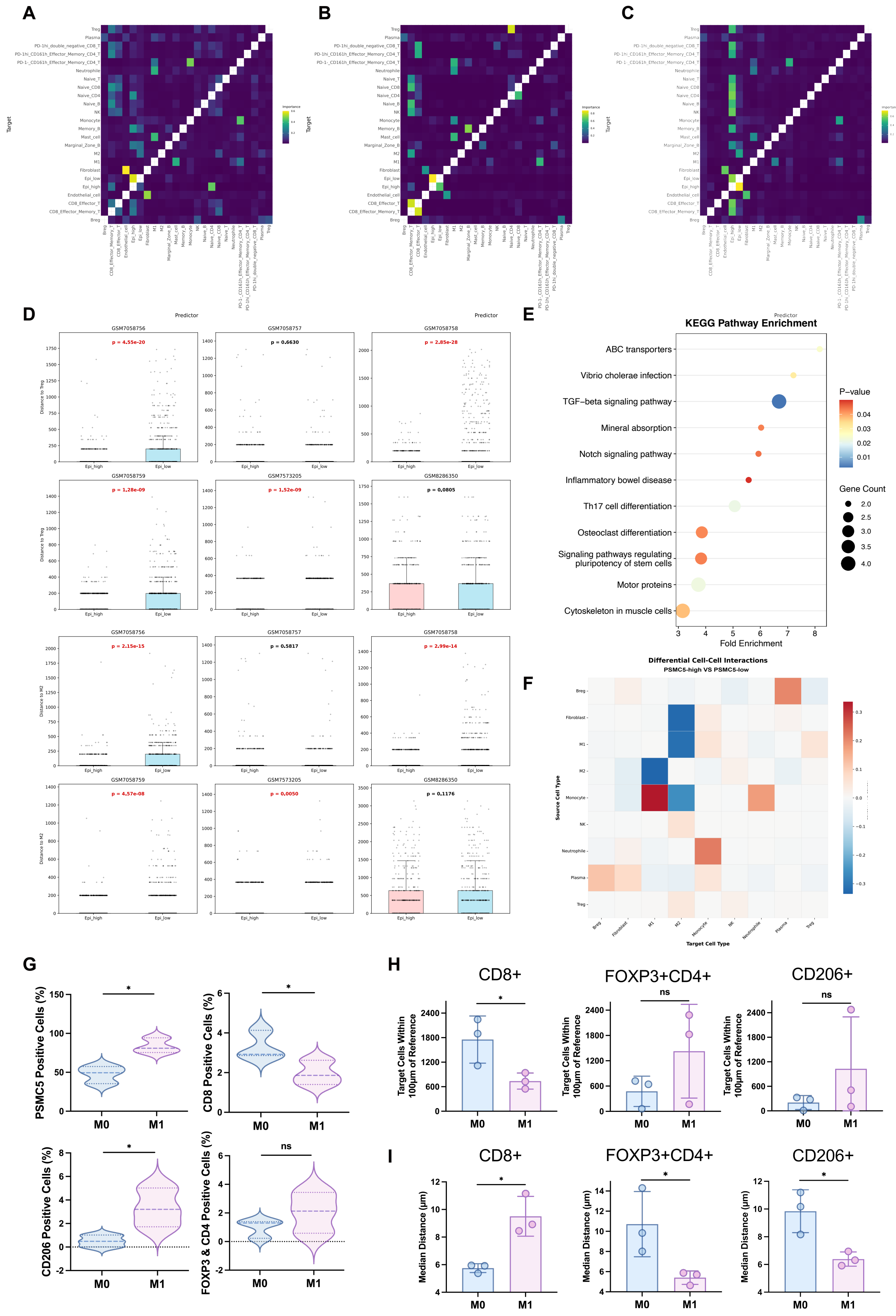
A
B
C
Target
Predictor
D
GSM7058756
GSM7058757
GSM7058758
GSM7058759
GSM7573205
GSM8286350
p = 4.55e-20
p = 0.6630
p = 2.85e-28
p = 1.28e-09
p = 1.52e-09
p = 0.0805
p = 2.15e-15
p = 0.5817
p = 2.99e-14
p = 4.57e-08
p = 0.0050
p = 0.1176
Distance to Treg
Distance to M2
Epi_high
Epi_low
E
KEGG Pathway Enrichment
ABC transporters
Vibrio cholerae infection
TGF−beta signaling pathway
Mineral absorption
Notch signaling pathway
Inflammatory bowel disease
Th17 cell differentiation
Osteoclast differentiation
Signaling pathways regulating pluripotency of stem cells
Motor proteins
Cytoskeleton in muscle cells
Fold Enrichment
P−value
Gene Count
F
Differential Cell-Cell Interactions
PSMC5-high VS PSMC5-low
Source Cell Type
Target Cell Type
Breg
Fibroblast
M1
M2
Monocyte
NK
Neutrophile
Plasma
Treg
G
PSMC5 Positive Cells (%)
CD8 Positive Cells (%)
CD206 Positive Cells (%)
FOXP3 & CD4 Positive Cells (%)
M0
M1
ns
H
CD8+
FOXP3+CD4+
CD206+
Target Cells Within 100μm of Reference
I
Median Distance (μm)

Figure S8 (A) Within-spot interaction heatmap summarizing co-occurrence of predictor and target cell types in the same Visium spot. (B) Adjacent-spot interaction heatmap depicting co-localization of predictor cell types in the six neighboring spots around each target cell type. (C) Paraspot interaction heatmap quantifying co-localization of predictor and target cell types in spots beyond the immediate six neighbors, indicating long-range association patterns within the tumor microenvironment. (D) Section-wise comparison of the average Euclidean distance between PSMC5-high or PSMC5-low epithelial spots and the nearest Treg or M2 macrophage spot across colorectal cancer and normal colonic tissue sections. Cell-type-specific spots were defined as the top 30% spots for each signature. (E) KEGG pathway enrichment analysis of genes upregulated in the PSMC5-high epithelial subpopulation compared with the PSMC5-low epithelial subpopulation. Bubble size indicates gene count, and color represents the enrichment P value. (F) Differential interaction heatmap comparing PSMC5-high versus PSMC5-low regions. Colors indicate increased (red) or decreased (blue) spatial association between source and target cell types. (G) Quantification of $PSMC5^{+}$cells, $CD8^{+}$ cells, $CD206^{+}$ cells, and $FOXP3^{+}CD4^{+}$ cells in primary colorectal tumors without metastasis (M0) and with metastasis (M1). (H) Quantification of $CD8^{+}$, $FOXP3^{+}CD4^{+}$, and $CD206^{+}$ cells within 100 μm of the reference region in M0 and M1 primary tumors. (I) Median distance from the reference region to $CD8^{+}$, $FOXP3^{+}CD4^{+}$, and $CD206^{+}$ cells in M0 and M1 primary tumors. Statistical analysis: hypergeometric test for (E); unpaired two-tailed Student's t test for (G-I). Data are presented as mean ± SD. For (D), P values are shown in the corresponding panels. *P<0.05, ns, not significant.

Variables	All patients (*n* = 146)	High (*n* = 73)	Low (*n* = 73)	*p* value
Age, n (%)				0.19
< 65	74 (51%)	33 (45%)	41 (56%)	
>= 65	72 (49%)	40 (55%)	32 (44%)	
Gender, n (%)				0.18
Female	64 (44%)	36 (49%)	28 (38%)	
Male	82 (56%)	37 (51%)	45 (62%)	
Size, n (%)				0.12
< 10 cm^3	51 (35%)	21 (29%)	30 (41%)	
>= 10 cm^3	95 (65%)	52 (71%)	43 (59%)	
T stage, n (%)				**< 0.001**
1	5 (3.4%)	3 (4.1%)	2 (2.7%)	
2	31 (21%)	5 (6.8%)	26 (36%)	
3	62 (42%)	36 (49%)	26 (36%)	
4	48 (33%)	29 (40%)	19 (26%)	
N stage, n (%)				**0.024**
0	86 (59%)	37 (51%)	49 (67%)	
1	39 (27%)	20 (27%)	19 (26%)	
2	21 (14%)	16 (22%)	5 (6.8%)	
M stage, n (%)				**0.007**
0	127 (87%)	58 (79%)	69 (95%)	
1	19 (13%)	15 (21%)	4 (5.5%)	
Stage, n (%)				**< 0.001**
I	26 (18%)	2 (2.7%)	24 (33%)	
II	57 (39%)	32 (44%)	25 (34%)	
III	44 (30%)	24 (33%)	20 (27%)	
IV	19 (13%)	15 (21%)	4 (5.5%)	
Vascular Invasion, n (%)				0.59
No	103 (71%)	50 (68%)	53 (73%)	
Yes	43 (29%)	23 (32%)	20 (27%)	
Nerve Invasion, n (%)				0.51
No	121 (83%)	59 (82%)	62 (85%)	
Yes	25 (17%)	14 (19%)	11 (15%)	
Lymph Node Status, n (%)				0.089
No	90 (62%)	40 (55%)	50 (68%)	
Yes	56 (38%)	33 (45%)	23 (32%)	

Table S1. Clinicopathological features associated with PSMC5 expression in colorectal cancer patients. P values were calculated using Fisher's exact test.

Variables	All patients (*n* = 146)	High (*n* = 73)	Low (*n* = 73)	*p* value
Age, n (%)				0.51
< 65	74 (51%)	39 (53%)	35 (48%)	
>= 65	72 (49%)	34 (47%)	38 (52%)	
Gender, n (%)				0.10
Female	64 (44%)	27 (37%)	37 (51%)	
Male	82 (56%)	46 (63%)	36 (49%)	
Size, n (%)				0.39
< 10 cm^3	51 (35%)	28 (38%)	23 (32%)	
>= 10 cm^3	95 (65%)	45 (62%)	50 (68%)	
T stage, n (%)				0.047
1	5 (3.4%)	3 (4.1%)	2 (2.7%)	
2	31 (21%)	22 (30%)	9 (12%)	
3	62 (42%)	26 (36%)	36 (49%)	
4	48 (33%)	22 (30%)	26 (36%)	
N stage, n (%)				**0.008**
0	86 (59%)	52 (71%)	34 (47%)	
1	39 (27%)	15 (21%)	24 (33%)	
2	21 (14%)	6 (8.2%)	15 (21%)	
M stage, n (%)				0.22
0	127 (87%)	66 (90%)	61 (84%)	
1	19 (13%)	7 (9.6%)	12 (16%)	
Stage, n (%)				**0.001**
I	26 (18%)	21 (29%)	5 (6.8%)	
II	57 (39%)	30 (41%)	27 (37%)	
III	44 (30%)	15 (21%)	29 (40%)	
IV	19 (13%)	7 (9.6%)	12 (16%)	
Vascular Invasion, n (%)				0.86
No	103 (71%)	52 (71%)	51 (70%)	
Yes	43 (29%)	21 (29%)	22 (30%)	
Nerve Invasion, n (%)				0.12
No	121 (83%)	64 (88%)	57 (78%)	
Yes	25 (17%)	9 (12%)	16 (22%)	
Lymph Node Status, n (%)				**0.006**
No	90 (62%)	53 (73%)	37 (51%)	
Yes	56 (38%)	20 (27%)	36 (49%)	

Table S2. Clinicopathological features associated with METTL14 expression in colorectal cancer patients. P values were calculated using Fisher's exact test.

Variables	All patients *(n = 146)*	High *(n = 73)*	Low *(n = 73)*	*p* value
Age, n (%)				0.51
< 65	74 (51%)	35 (48%)	39 (53%)	
>= 65	72 (49%)	38 (52%)	34 (47%)	
Gender, n (%)				0.74
Female	64 (44%)	33 (45%)	31 (42%)	
Male	82 (56%)	40 (55%)	42 (58%)	
Size, n (%)				0.22
< 10 cm^3	51 (35%)	22 (30%)	29 (40%)	
>= 10 cm^3	95 (65%)	51 (70%)	44 (60%)	
T stage, n (%)				**<0.001**
1	5 (3.4%)	3 (4.1%)	2 (2.7%)	
2	31 (21%)	6 (8.2%)	25 (34%)	
3	62 (42%)	34 (47%)	28 (38%)	
4	48 (33%)	30 (41%)	18 (25%)	
N stage, n (%)				**<0.001**
0	86 (59%)	23 (32%)	63 (86%)	
1	39 (27%)	32 (44%)	7 (9.6%)	
2	21 (14%)	18 (25%)	3 (4.1%)	
M stage, n (%)				0.027
0	127 (87%)	59 (81%)	68 (93%)	
1	19 (13%)	14 (19%)	5 (6.8%)	
Stage, n (%)				**<0.001**
I	26 (18%)	1 (1.4%)	25 (34%)	
II	57 (39%)	21 (29%)	36 (49%)	
III	44 (30%)	37 (51%)	7 (9.6%)	
IV	19 (13%)	14 (19%)	5 (6.8%)	
Vascular Invasion, n (%)				0.1
No	103 (71%)	47 (64%)	56 (77%)	
Yes	43 (29%)	26 (36%)	17 (23%)	
Nerve Invasion, n (%)				0.83
No	121 (83%)	60 (82%)	61 (84%)	
Yes	25 (17%)	13 (18%)	12 (16%)	
Lymph Node Status, n (%)				**<0.001**
No	90 (62%)	27 (37%)	63 (86%)	
Yes	56 (38%)	46 (63%)	10 (14%)	

Table S3. Clinicopathological features associated with SMURF1 expression in colorectal cancer patients. P values were calculated using Fisher's exact test.

Antibodies used for western blot

Primary antibodies included PSMC5 (ab178681), SMURF1 (ab300408), METTL14 (26158-1-AP), N-cadherin (A19083), E-cadherin (ab231303), Snail (13099-1-AP), Twist (25465-1-AP), Vimentin (10366-1-AP), PLEKHO1 (24883-1-AP), GAPDH (60004-1-Ig), Ubiquitin (80992-1-RR), HA tag (51064-2-AP), FLAG tag (66008-4-Ig), MYC tag (60003-2-Ig), normal rabbit IgG isotype control (98136-1-RR) and Anti-Ubiquitin K11 linkage (MABS107-I).

Secondary antibodies were HRP-conjugated goat anti-mouse IgG (SA00001-1), HRP-conjugated goat anti-rabbit IgG (SA00001-2), and Universal type anti-heavy chain + light chain secondary antibody (M21008).

Table S4. Sequences of primers used for qRT-PCR.

METTL14-F	GGGGTTGGACCTTGGAAGAG
METTL14-R	CCCATGAGGCAGTGTTCCTT
N-cadherin-F	TGGAGCCTGATGCCATCAAG
N-cadherin-R	GAGCTGTGGGGTCATTGTCA
Vimentin-F	GGACCAGCTAACCAACGACA
Vimentin-R	AAGGTCAAGACGTGCCAGAG
Twist-F	GTCCGCAGTCTTACGAGGAG
Twist-R	GCCAGCTTGAGGGTCTGAAT
Snail-F	GAGGACAGTGGGAAAGGCTC
Snail-R	TGGAGATCCTTGGCCTCAGA
SOX4-F	GTCCCACTCCTCCTCTTCCT
SOX4-R	AGCCGGGCTCGAAGTTAAAA
E-cadherin-F	TTGAAGATTGCACCGGTCGA
E-cadherin-R	TCTGTAGGTGGAGTCCCAGG
GAPDH-F	GATTTGGTCGTATTGGGCGC
GAPDH-R	TTCCCGTTCTCAGCCTTGAC

Graphical abstract citation

Created in BioRender. Zhang, E. (2026) https://BioRender.com/hs8awlt
